# Maximizing genetic gain through unlocking genetic variation in different ecotypes of kalmegh (*Andrographis paniculata* (Burm. f.) Nee)

**DOI:** 10.3389/fpls.2022.1042222

**Published:** 2022-11-07

**Authors:** Trishna Chaturvedi, Anil Kumar Gupta, Karuna Shanker, Basant Kumar Dubey, Gunjan Tiwari

**Affiliations:** ^1^ Division of Plant Breeding and Genetic Resource Conservation, Central Institute of Medicinal and Aromatic Plants, Council of Scientific and Industrial Research, Lucknow, Uttar Pradesh, India; ^2^ Phytochemistry Division, Central Institute of Medicinal and Aromatic Plants, Council of Scientific and Industrial Research, Lucknow, Uttar Pradesh, India; ^3^ Biotechnology Division, Central Institute of Medicinal and Aromatic Plants, Council of Scientific and Industrial Research, Lucknow, Uttar Pradesh, India

**Keywords:** genetic diversity, agro-morphological, phytochemical, EST-SSR markers, *Andrographis paniculata*

## Abstract

*Andrographis paniculata*, commonly known as kalmegh is among the most popular medicinal herbs in Southeast Asia. It is widely cultivated for medicinal purposes. The bioactive molecule, Andrographolide accumulated in herb leaves has immense therapeutic and economic potential. However, comprehensive information regarding genetic diversity is very limited in this species. The present study assessed genetic diversity between and within the six populations (ecotypes) of twenty-four kalmegh accessions using multiple datasets (agro-morphological traits, phytochemical traits, and genic markers). This is the established report where EST-SSR (Expressed sequence tags-Simple Sequence Repeat) markers have been used to unlock genetic variation in kalmegh. Here, we identified and developed ninety-one metabolic pathway-specific EST-SSR markers. Finally, 32 random EST-SSR primer pairs were selected for genetic diversity assessment. Multivariate analysis to unveil the agro-morphological, phytochemical and genotypic variability was helpful in discriminating various germplasms studied in the present study. Among all the morphological discriptors used in present study, days to fifty percent flowering and dry herb yield were found as potential selection index for AP genetic improvement. Hierarchical cluster analysis built with agro-morphological data identified three major groups. However, corresponding analysis with phytochemical and molecular data generated two clear-cut groups among the studied individuals. Moreover, the grouping of individuals into different clusters using multiple datasets was geographically independent, and also showed inconsistency in grouping among agromorphological, phytochemical and molecular dataset based clusters. However, joint analysis using agro-morphological, phytochemical and genotypic information generated two genetic groups, which could be a valuable resource for identifying complementary crossing panels in the kalmegh breeding program. The accessions AP7, AP13, AP5, AP3 belong to cluster I and accessions AP17, AP18 belong to cluster II could be utilized as potential donors for high dry herb yield and andrographolide content, respectively in different selective breeding programs of AP. Thus, our results provided useful information about the overall genetic diversity and variation in economic traits useful for initiating selective breeding programs for contrasting traits of interest and maximizing genetic gain in kalmegh.

## 1 Introduction

Integrated approaches are required to accelerate the genetic gain over time in crop improvement programs concerning various economic traits and to realize the improved gain in farmers’ fields. One of the critical components required to maximize genetic gain is the enhanced genetic variance (σ^2^g) which can be achieved by unlocking or creating desirable alleles/genotypes and deploying them for improving key traits. It is desirable to assess new alleles for target traits and introgress them in breeding populations while maintaining genetic diversity. Comprehensive approaches involving phenotyping, chemotyping, genotyping, and bioinformatics tools present an enormous opportunity to measure novel alleles precisely and review the breeder’s equations for genetic improvement over time.

Kalmegh (*Andrographis paniculata* (Burm. f.) Wall. ex Nees.) is a self-pollinated, annual, diploid (2n=50), and highly traded medicinal herb of the family Acanthaceae. It is well known with other vernacular names like Green Chirata, bhui-neem, king of bitter, etc. There are roughly 40 species in the genus *Andrographis*, with kalmegh (*Andrographis paniculata*) being the most popular medicinal plant species (Boopathi, 2000) of the genus. It is said to have originated in the southern parts of India and Sri Lanka and has a broad geographical distribution in tropical and subtropical regions of the country. Several bioactive specialized metabolites have been identified from *Andrographis paniculata* (AP), including ent-labdane-related diterpenes (ent-LRDs), phenylpropanoids, xanthones, and flavonoids (Koteswara et al., 2004; Li et al., 2007). Ent-LRDs that accumulated in the leaves and thought to be the major bioactive ingredients of kalmegh are Andrographolide (AG), Neoandrographolide (NAG), 14-deoxy-11,12-didehydroandrographolide (DDAG) and Andographanin (AN) (Ooi et al., 2011; Valdiani et al., 2012; Mondal et al., 2013; Lin et al., 2014; Raghavan et al., 2014; Wang et al., 2014) among which, Andrographolide is the most prevalent ones and has been widely researched for various pharmacological activities (Chopra, 1956; Akbar, 2011; Garg et al., 2015; Chauhan et al., 2019). The andrographolide based drugs are reported to have numerous biological activities such as hepatoprotective, anti-diabetic, anti-oxidant, immune-modulatory, anti-allergic, anti-pyretic, antidiarrhoeal, and anti-HIV activity (Garg et al., 2015). Recent experiments have also shown its anti-cancerous activity in human cancer cells (Luo et al., 2014). Andrographis paniculata has blood purifying action and is suggested for curing gonorrhea, leprosy, and several skin disorders (Pandey and Rao, 2018). The herb derived from leaves or aerial parts of the Kalmegh is known as Chuaxinlian, Lanhelian, or Yijianxi in the Chinese system of medicine. It possesses similar properties as described in the traditional system of medicine in India. Various preparations and formulations of the Kalmegh have been used to treat infectious and non-infectious diseases with significant efficacy reported in case of epidemic encephalitis B, vaginitis, pelvic inflammation, herpes zoster, neonatal subcutaneous annual ulcer, chickenpox, mumps, neurodermatitis, eczema, and burns (Lim et al., 2012). Besides andrographolides, flavonoids, caffeic acid, and chlorogenic acid are also produced in this plant (Rao et al., 2004). Kalmegh is reported to show high efficacy against chronic malaria and is often used as an alternative to *Swertia chirata* (Valdiani et al., 2012). Recent *in-silico* analysis suggested the potential role of Andrographolide against SARS-CoV-2 main protease (Mpro) (Enmozhi et al., 2020). Thus, *Andrograhis paniculata* (AP) has immense therapeutic and economic potential. Quality dry herbs of the plant are sold for as much as Rs. 17-30/kg (source: e-Charak, 2008). The costs of Andrographis powder, with varied diterpenoid content, ranged from US$0.12 per gram to US$ 0.70 per gram in July 2016. Also, the price offered by Sigma-Aldrich for 100 and 500gm packages of pure Andrographolide (98%) was 44.2USD and 162.50 USD, respectively, in the same year (Pandey and Rao, 2018). However, there is a considerable gap between the demand and supply of quality raw herbs on national and international platforms. The heavy demand for diterpene andrographolide has motivated Indian farmers to commercialize kalmegh cultivation. However, to meet global needs, most raw herbs are rigorously collected from wild habitats causing massive mutilation of genetic diversity and shifting this species on the verge of extinction. Growing kalmegh under captive cultivation is the only way to prevent the loss of natural diversity from the wild source and meet global demand. Plant breeding and biotechnology are potential tools to bring kalmegh into captive cultivation, which entails great genetic variations and maximizes genetic gain by utilizing more selection programs effectively. Although few commercial cultivars are available, they are ecotype specific, minimal, and insufficient to meet national and international demands. The major bottleneck for the resulting yield gap is the narrow genetic base of the existing cultivars. Sustainable yield increase can only be achieved by introducing new sources of favorable alleles from different ecotypes into the rapid breeding cycle and attaining kalmegh production challenges.

Systematic evaluation and cataloging of genetic diversity at morphological and phytochemical levels are extremely useful for effective conservation and optimum genetic amelioration of allelic and genotypic variability. In the recent past, the introgression of molecular markers has augmented the accumulation of genetic gains achieved by morpho-chemical descriptors based characterization. In kalmegh, an array of genetic diversity studies has been done using agro-morphological traits (Nagvanshi and Tirkey, 2016), phytochemical traits (Sabu et al., 2001; Archana et al., 2016), and molecular markers, including RAPD, ISSR, SCoT, CBDP and Genomic SSRs (Padmesh et al., 1999; Maison et al., 2005; Kumar and Shekhawat, 2009; Wijarat et al., 2011; Ghosh et al., 2014; Tiwari et al., 2016; Kumar et al., 2020). Compared with dominant markers, the reproducibility and reliability of SSR (simple sequence repeat) markers are high due to co-dominance, high polymorphism, uniform distribution throughout the genome, and multi-allelic nature (Varshney et al., 2005). However, the number of SSR markers reported in kalmegh is limited, and most of them are genomic SSRs, the construction of which is a tedious and costly affair.

In contrast, the advent of modern genomics-based *denovo* transcriptome assembly in kalmegh has rapidly paved the way to mine and develop a large number of high throughput unigene-based SSRs (EST-SSRs) at low cost. These SSRs are possibly linked with particular transcriptional regions that contribute to agronomic traits and are well suited for marker-assisted breeding in *Andrographis paniculata*. To date, EST-SSR based genetic characterization has not been done in AP. Moreover, comprehensive data regarding genetic diversity studies are scanty and provide the rationale for this study (Lattoo et al., 2008; Sharma et al., 2009; Valdiani et al., 2014; Hiremath et al., 2020). This information would be essential to exploit beneficial genes present in indigenous genetic resources of different ecotypes to increase the selection efficiency in kalmegh breeding and for adequate biodiversity protection and management. Moreover, it would also be intriguing to see if there is any conceptual or empirical agreement between agro-phytochemical features and molecular markers to accelerate kalmegh breeding and maximize genetic gain. Thus, with this backup, the present study was designed to (i) evaluate the genetic diversity of various germplasm of kalmegh at agro-morphological and phytochemical levels to identify superior individuals/genotypes for the breeding purpose (ii) assess molecular diversity and population structure by employing metabolic pathways specific EST-SSRs and (iii) finally comparison and joint analysis of agro-phytochemical traits and molecular information to provide in-depth insight into the genetic variability present in studied germplasm.

## 2 Materials and methods

### 2.1 Plant materials

**Figure 1:**
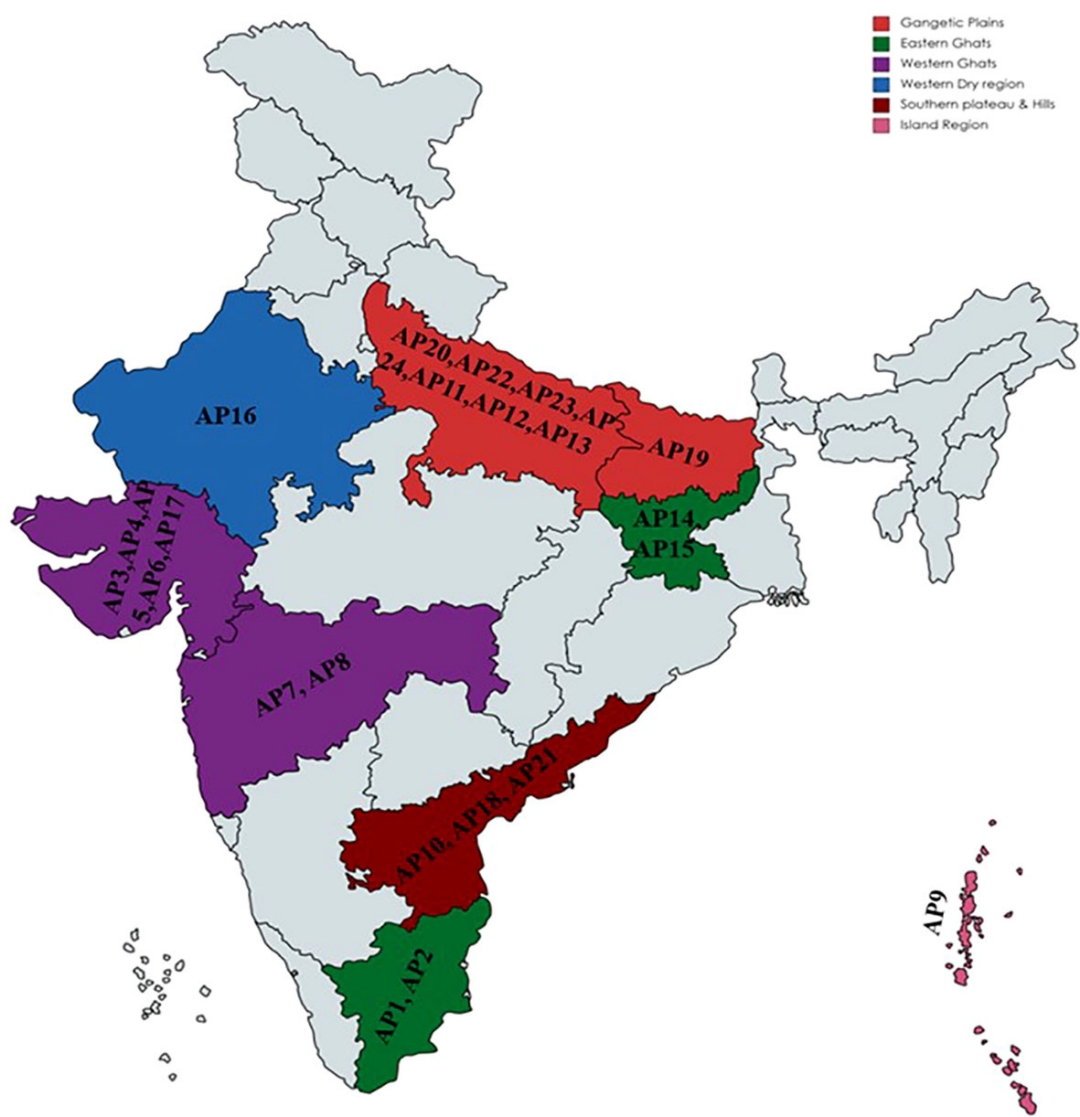
Geographical distribution of twenty-four kalmegh accessions used in the present study.

The experimental material covered twenty-four diverse accessions of Kalmegh (*A. paniculata)*, including one released and cultivated variety as a local check (**Supplementary Table S1**). All the accessions were procured from different states of India, covering six agroecological regions of the country (**Figure 1**), and conserved and maintained over the years at the National Gene bank of CSIR-Central Institute of Medicinal and Aromatic Plants (CIMAP), Lucknow (India).

### 2.2 Experimental designs

The present study was initiated at the Field Research Centre of CSIR- CIMAP, Lucknow, India (80.50°C E longitude and 26.5°C N latitude), where the annual temperature varies between 5°C to 45 °C. For the genetic diversity study, nursery planting was done under outdoor conditions using individual open-pollinated seed lots of twenty-four accessions in earthen pots having a combination of sandy loam soil and vermicompost in the ratio of 4:1 in the month of June 15^th^, 2020, and June 15^th^, 2021. After 45 days of nursery germination, transplanting was done into 4.5 x 3.5m plots in a randomized complete block design with 3 replications at 35 x 30 cm spacing in both years. Standard cultivation practices were followed to raise healthy populations in both years, i.e., 2020 and 2021.

### 2.3 Evaluation of agro-morphological data

Nine agro-morphological descriptors were measured at the reproductive stage [120-130 days after transplanting (DAT)]. Data on days to 50% flowering (DFF) and days to maturity (DM) was scored on a plot basis. However, in each replication, ten competitive plants of each accession were selected to measure other traits, i.e., plant height (PH) (cm), Number of nodes per plant (NNP), Number of secondary branches per plant (NSBP), leaf length (LL) (cm), leaf width (LW) (cm), Inflorescence length (IL) (cm), and dry herbage yield per plant (DHY) (g)

### 2.4 Extraction of Ent-LRDs and high-performance liquid chromatography (HPLC) analysis

Aerial parts of the plant of each accession were picked at the maturity stage and were shade dried at room temperature for seven days. Dried samples were ground into a fine powder and stored under -20°C for a short period. About 100mg of powdered samples were extracted three times in 10ml of analytical grade methanol on a sonicator at 30mins intervals. All the filtrates were combined to obtain a total volume of 30ml and evaporated to dryness under a water bath. For HPLC analysis, the dried filtrate of each sample was dissolved in 1ml HPLC grade methanol (Merck, Germany). The extraction process for each accession was done using five biological replicates.

A 20ml solvent extract of individual accession was filtered into HPLC vials using disposable polypropylene syringe filters and injected into the HPLC-UV (Shimadzu LC-10A, Tokyo, Japan) system. The analysis was carried out as defined earlier by Tewari et al., 2010. The stock solutions of the standard compounds of AG, NAG, DDAG, and AN were also prepared in HPLC grade methanol at a concentration of 1mg/ml for standard curve preparation and quantifying of the experimental samples.

### 2.5 DNA extraction and genotyping with EST-SSR markers

The genomic DNA of each accession was isolated from 0.2g frozen leaves with a modified CTAB (Cetyl triethyl ammonium bromide) extraction method (Khanuja et al., 1998) (**Supplementary Table S2**). DNA quality was checked, and quantity was estimated using 0.8% agarose gel, and nanodrop (Thermo Fisher, USA) at 260/280 and 260/230 OD ratios, respectively. After that, a working concentration of 10ng/µL was made for each accession and kept at 4°C for further use.

The 53 non-redundant combined master control transcripts annotated and reported to be involved in specialized metabolic pathways in kalmegh variety “CIM-Megha” by Garg et al. (2015) were rescanned for the presence of SSR motifs using the Batch Primer3 v1.0 tool (http://probes.pw.usda.gov/cgibin/batchprimer3/batchprimer3.cgi) developed by You et al. (2008). Similar repeat motifs were identified using this tool, as reported earlier by Garg et al. (2015). The in-built Primer3 core program of Batch Primer3 v1.0 was used to design primer pairs using flanking sequences. Out of 53, a total of 91 SSR primers were identified from 50 master control transcripts. To test the polymorphism of EST-SSR primers in kalmegh,32 primer pairs were randomly selected. The target amplicon size was set as 100-300bp, melting temperature (T^M^) as 50-60°C, GC percentage as 40-60%, and optimal primer length between 18-25bp.

PCR reactions were carried out in a10µL volumes containing 5µL of dye mixed with One PCR™ supermix (GeneDirex, Taiwan), 0.5µL of forward primer (5pmol), 0.5µL of reverse primer (5pmol), and 40ng of template DNA. PCR reactions were performed in a Thermal Cycler (Bio-Rad, California, USA) using the following cycling conditions: initial denaturation for 5 min at 94°C followed by 35cycles of denaturation for 1min at 94 °C, annealing for 1min at temperatures ranging between 50.5 °C to 57.8 °C depending upon the primer T^M^ and extension for 2mins at 72 °C followed by final extension for 7mins at 72 °C. PCR products were stored at 4 °C for gel electrophoresis. Metaphor agarose gel (2%) was used to separate amplified products using a power supply of 80V for 2.5-3h and visualized under Gel documentation System (UVP Bioimaging system, Analytik-Jena, Germany). DNA ladder of 100bp (Gene Direx, Taiwan) was used as standard.

### 2.6 Statistical analysis of agro-morphological and phytochemical data

All the quantitative data were statistically analyzed in software R-studio v4.0.3. The ‘variability’ package was used to perform an analysis of variance (ANOVA) on mean values of agro-morphological traits across replications (Popat et al., 2020). Pooled data of both years were used to analyze various genetic parameters such as Genotypic and Phenotypic coefficient of variation (Singh & Chaudhary, 1985), heritability, and genetic advance as percent of the mean (Johnson et al., 1955) using the same package in R-studio. The principal component analysis (PCA) for both types of traits was done in the ‘pca3d’ package of R software (Weiner and Weiner, 2016). To identify the divergence among different ecotypes, agglomerative hierarchical clustering methods were employed to create a tree diagram using ‘ggplot2’ package (Wickham et al., 2016). The K-mean methods of function ‘factoextra’ were applied to determine the number of k-groups to explain the agronomic and chemical variation among tested populations. Calculating pair-wise Euclidean distance and cluster analysis was performed using function ‘NbClust’ of package ‘ggplot2’. Further, a heatmap was also prepared to visualize data more clearly using the function ‘heatmap.2’ in package ‘gplots.’

### 2.7 Analysis of genotyping data

A binary data matrix was prepared to perform molecular data analysis by scoring the presence (1) or absence (0) of amplified bands produced after gel electrophoresis. The discriminating power of primers was assessed by measuring four parameters; PIC (Polymorphic Information Content), Rp (Resolving Power), MI (Marker Index), and EMI (Effective Marker Index). The PIC was calculated following Botstein et al. (1980) as: PIC=1-∑fi^2^, where, ‘fi’ = frequency of the ‘i^th^’ allele (band present). Similarly, the resolving power of each primer was measured following Prevost and Wilkinson (1999) method as: Rp= Ʃ Ib=1 − [2 × |0.5 − p|], where, Ib= band informativeness and p= number of individuals containing band. Further, MI provides a convenient estimate of marker utility and is calculated as: MI=EMR x PIC, where EMR (Effective multiplex ratio)= number of polymorphic band × fraction of polymorphic band (Milbourne et al., 1997; Prevost and Wilkinson, 1999). EMR determines the number of polymorphic loci analyzed per experiment in the germplasm set of interest. Varshney et al. (2007) provided an index called the Effective marker Index (EMI) to accelerate the practicability of the marker system to plant breeders. EMR is calculated as: EMR=MI x QND or, EMR= MI x DC x QM x PR, where QND= Qualitative nature of data, DC=Documentation capability, QM= Quality of Marker, and PR= Percent Reproducibility of the band/fragment of the marker. DC and PR represent the constant value and are set as 0.75 and 1.0 for SSR markers, respectively. However, the QM value varies with different primer pairs and is defined as per the scale (0.25 to 1.0) given by Varshney et al. (2007). Here, we also measured the EMI of all the polymorphic markers and took a scale of 1.0 as a QM value to estimate QND since all the amplified bands were single and clear.

The genetic similarity matrix and phenetic analysis of binary datasets were performed by software NTSYS v2.02e (Rohlf, 2000). Genetic relatedness among the twenty-four kalmegh accessions was estimated using the SIMQUAL module of Jaccard’s similarity coefficient (Jaccard, 1908). The UPGMA (Un-weighted Pair Group Method with Arithmetic Mean) algorithm along with the SAHN (Sequential agglomerative hierarchical and nested clustering method) module of the same software was also used to compute a dendrogram demonstrating genetic association among all the accessions. Moreover, a Model-based population structure study was carried out to study the genetic association in the twenty-four accessions of kalmegh using polymorphic EST-SSR primers. STRUCTURE software version 2.3.4 (Pritchard et al., 2000) was used to perform this analysis. The analysis was performed without incorporating the population information and considering both the admixture model and correlated allele frequencies between the populations. Here, accessions from same ecotypes were considered as single populations, thereby forming six populations. The K values were set to 1-10, and the software was run three times (r=3) for each K (number of populations). The number of Markov Chain Monte Carlo (MCMC) replications and burn-in-period was set to 100,000 for each run for all the twenty-four accessions to evaluate the number of populations. The plateau of the ΔK values was plotted using Ln(PD), which was derived for each K (Evanno et al., 2005). The online program “structure harvester” was used (http://taylor0.biology.ucla.edu) to compute the final number of K in population structure. The analysis of molecular variance (AMOVA) and Principal Coordinate Analysis (PCoA) was performed using GenAlEx6.501 software (Peakall and Smouse, 2012) to partition the genetic variation in studied kalmegh accessions. A Mantel test was also done to reveal the correlation between phytochemical and genotypic distance matrices using the same software. Finally, the dendextend R package was used to assess the correlation between two dendrograms generated for agro-phytochemical and molecular datasets (Tal, 2015). A joint cluster analysis was also executed by combining the distance matrices of all datasets generated in the present study using the R package (Garnier et al., 2018).

## 3 Results

The present study conducted with twenty-four accessions of *A. paniculata* were collected from thirteen states and belong to India’s six ecotypes (agro ecological regions) (**Figure 1**). Out of twenty-four, 23 (AP1 to AP23) were germplasm accessions, and one was a cultivated variety, CIM-Megha (AP24) (**Supplementary Table S1**). To unlock the genetic variation present in *A.paniculata* accessions, three different tools, including phenotypic, phytochemical, and molecular markers (EST-SSRs) were used.

### 3.1 Agro-morphological diversity

**Figure 2:**
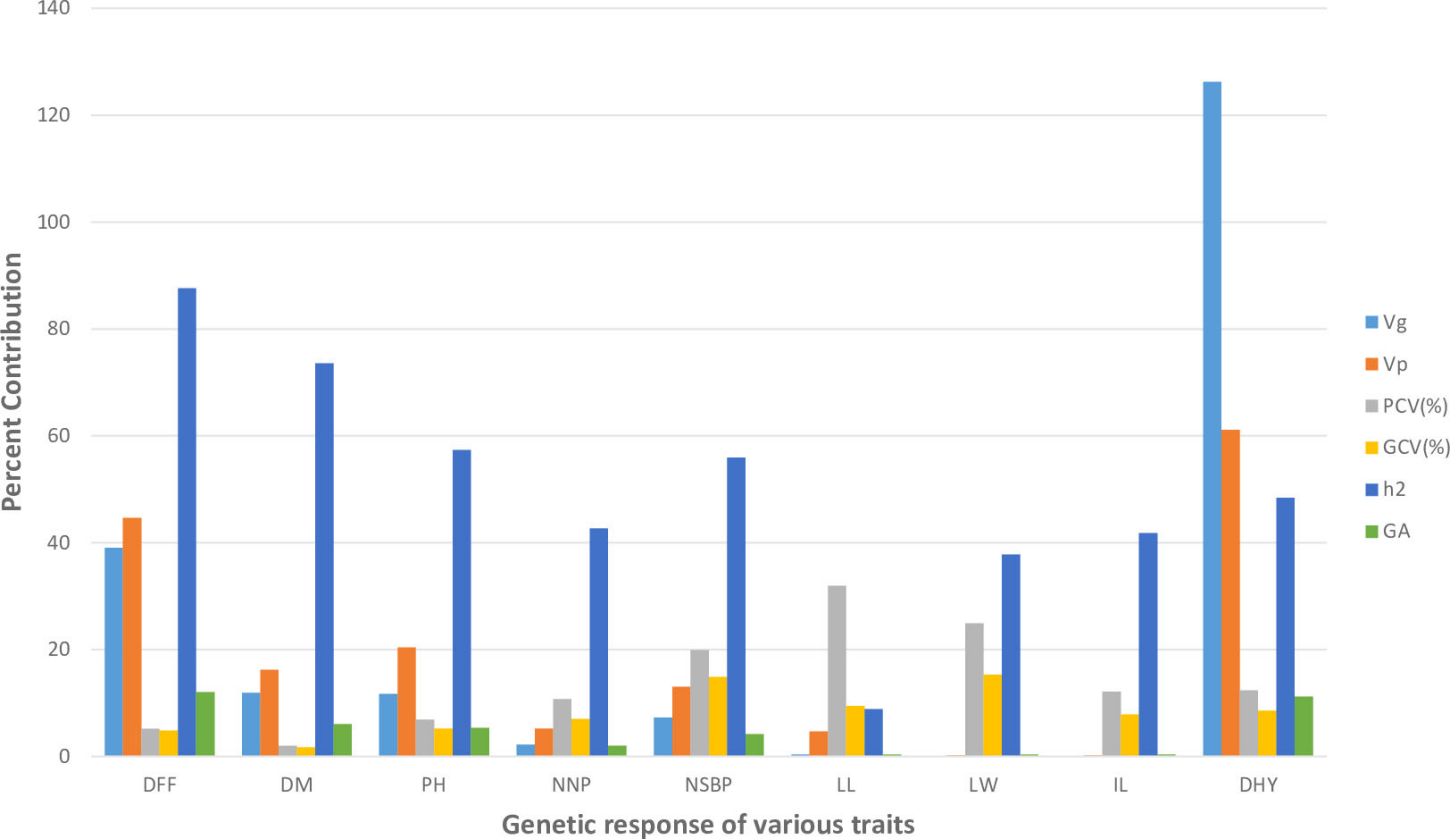
Graphical representation of genetic variability parameters estimated for nine quantitative traits in the present study.

The phenotypic diversity was assessed among experimental sets using nine agro-morphological (quantitative) traits. The analysis of variance (ANOVA) results are mentioned in **Supplementary Table S3(A)**. The ANOVA results showed substantial variation among all the accessions for the maximum number of traits studied except leaf length. The results of the mean comparison and genetic variability parameters for all studied traits are mentioned in **Supplementary Tables S3(B) and S3(C)**. For all the metric traits studied (**Figure 2**), the PCV (phenotypic coefficient of variation) was consistently greater than the GCV (genotypic coefficient of variation), and ranged from 2.03% (DM) to 31.92% (LL). However, the estimates of GCV varied from 1.74%(DM) to 15.33% (LW). The selection efficiency parameters, heritability ( ${\text{h}}_{\text{bs}}^{2}$ ) and genetic advance (GA) ranged from 8.86% (LL) to 87.59% (DFF) and 0.37% (LW) to 12.05% (DFF), respectively. The highest heritability with moderate genetic advance was estimated for days to fifty percent flowering (DFF) trait. However, moderate heritability with moderate genetic advance was observed for dry herb yield (DHY).

**Figure 3:**
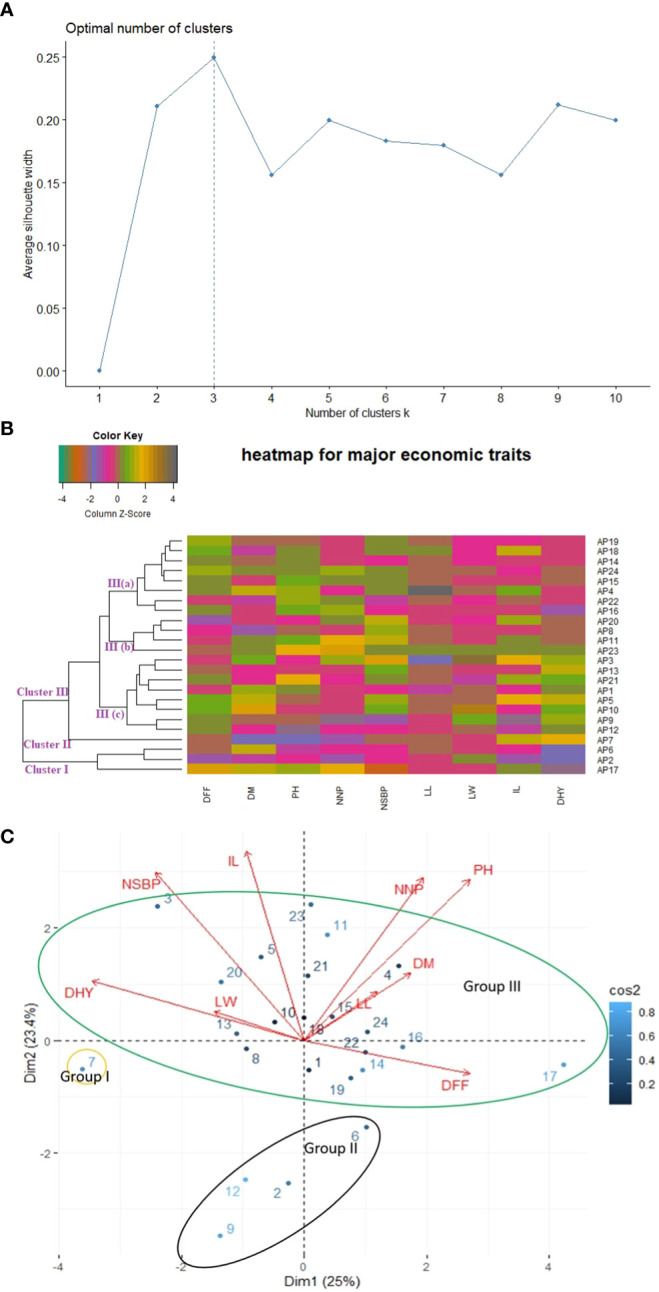
Relationship among twenty-four accessions of kalmegh based on agronomic traits using **(A)** k-mean **(B)** Euclidean distance **(C)** PCA biplot.

Different clustering methods were used to execute phenetic analysis considering agro-morphological traits to gain reliable and precise estimates of genetic diversity present in the experimental sets. K-mean clustering, Euclidean distance-based agglomerative hierarchically clustered heatmap, and Eigen value-based Principal Component Analysis (PCA) divided all the studied germplasm into three clusters (**Figures 3A–C**). However, the grouping of individuals into different clusters was entirely different. The results of Euclidean distance-based clustering are shown in **Figure 3B**. Three accessions (AP6, AP2, and AP17) were included in Cluster I. These accessions were grouped since they had a similar range of dry herb yield and leaf length. Out of the three accessions, two were from ecotype Western Ghats (WG), and one was from ecotype Eastern Ghats (EG). In Cluster II, accession AP7 was grouped alone. This accession was superior to other accessions concerning dry herb yield and days to fifty percent flowering and belonged to the WG ecotype. Cluster III was observed as the largest group on the heat map and divided into 3 sub-groups III(a), III(b), and III(c). Sub-group III(a) covered eight accessions and was observed to be superior for plant height but not for dry herb yield. Among eight accessions, three (AP22, AP24, and AP 19) were associated with Gangetic Plains (GP), two (AP15 and AP14) to EG, one individually to WG (AP4), Western dry regions (WDR) (AP16) and Southern Plains and Hills (SP&H) (AP18). Likewise, Cluster III (b) grouped four accessions in which three (AP23, AP11, and AP20) were related to ecotype GP and one to ecotype WG (AP8). All the accessions of Cluster III(b) were superior to other accessions for the number of secondary branches per plant and found in a favorable position for dry herb yield. Cluster III(c) also grouped eight accessions, of which two belong to ecotype GP (AP12 & AP13), two separately to WG (AP3 and AP5), and SP&H ecotypes (AP10 and AP21), one individually to EG(AP1) and Island Region (IR) ecotype (AP9). All the accessions of Cluster III (c) were found superior to other accessions for dry herb yield except accession AP7, which showed the highest dry herb yield compared to all other accessions.

PCA clustered accessions into different groups based on their eigenvalues. The PCA results showed that the first two principal components (PC1 and PC2) collectively elucidated 48.40% of the total variation, as mentioned in **Supplementary Table S4(A)**. Based on results shown in **Figure 3C**, the first area of the biplot, which included positive values of both components, covered accessions AP23, AP11, AP21, AP18, AP15, AP4, and AP24 and associated with traits like the number of nodes per plant, days to maturity, plant height, and leaf length. Likewise, the second area of the biplot, which comprised positive values of the second component and negative values of the first component, positioned accessions AP1, AP6, AP22, AP19, AP14, AP16, and AP17 on the biplot and associated with days to fifty percent flowering trait. The third area of the biplot, which has negative values for both components, covered five accessions (AP2, AP7, AP8, AP9, and AP12), and no significant association with studied traits was observed. Lastly, in the fourth area of the biplot, which contained a positive value of the PC1 and a negative value of the PC2, accessions AP3, AP5, AP10, AP13, and AP20 were located and linked with dry herb yield, number of secondary branches/plant, leaf width, and inflorescence length. Overall, all accessions were classified into three clear-cut groups on the biplot display and clustered with four accessions in group I, one accession in group II and nineteen accessions in group III, somewhat similar to cluster analysis.

### 3.2 Phytochemical diversity

**Figure 4:**
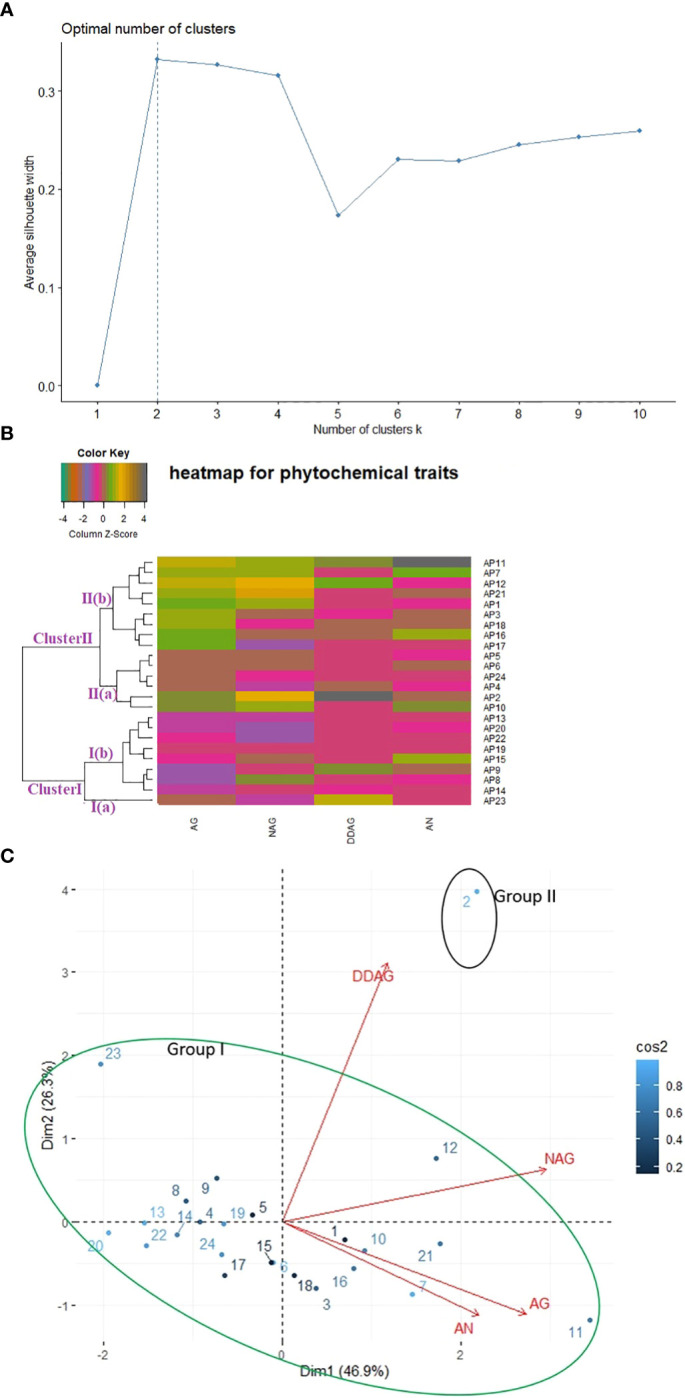
Relationship among twenty-four accessions of kalmegh based on phytochemical traits using **(A)** k-mean **(B)** Euclidean distance **(C)** PCA biplot.

A phytochemical dataset of twenty-four accessions was also used to estimate the level of variability present among them (**Supplementary Table S4B**). K-mean clustering divided the whole accessions into two clusters, confirmed further by agglomerative hierarchical clustering based heatmap and PCA (**Figures 4A–C**). The one-way heatmap clustered the whole germplasm into two main groups (**Figure 4B**): Cluster I covered nine accessions, and Cluster II comprised fifteen accessions. Cluster I was divided further into two sub-clusters- Sub-cluster I(a) and Sub-cluster I(b). The accession AP23 was grouped alone in Sub-cluster I(a) and found in a favorable position for 14-deoxy-11,12-didehydro-andrographolide (DDAG) content. Sub-cluster I(b) consisted of eight accessions (AP14, AP8, AP9, AP15, AP9, AP22, AP20, and AP13) and showed no significant association with any phytochemical trait. Likewise, Cluster II was further divided into two sub-clusters- Sub-cluster II(a) and Sub-cluster II(b) and showed superior association with andrographolide content (AG) compared to other studied accessions. Sub-cluster II (a) grouped six accessions (AP2, AP4, AP5, AP6, AP10, and AP24) and Sub-cluster II (b) covered nine accessions (AP1, AP3, AP7, AP11, AP12, AP16, AP17, AP18, and AP21).

In PCA, the first two principal components (PC1 and PC2) collectively described 73.28% of the total variability, as shown in **Supplementary Table S4(C)**. As per the PCA biplot (**Figure 4C**), the first area of the biplot covered two accessions (AP2 and AP12) and showed an association with DDAG and neoandrographolide (NAG) content. However, eight accessions (AP1, AP3, AP7, AP10, AP11, AP16, AP18, and AP21) were grouped in the second area of the biplot and encompassed Andrographolide (AG) and Andrographanin (AN) content. The third and fourth areas of the biplot included ten (AP4, AP6, AP13, AP14, AP15, AP17, AP19, AP20, AP22, and AP24), and three (AP5, AP8, AP9) accessions, respectively, and showed no significant association with the studied phytochemical trait. Overall, on the PCA biplot, two groups were visible, encompassing maximum accessions in group I and single accession (AP2) in group II.

### 3.3 Molecular diversity

#### 3.3.1 SSR primer designing

In total, 91 primer pairs were designed from 50 unique transcripts (ESTs), 23 (46%) of which comprised more than one SSR loci, as shown in **Supplementary Table S5(A)**. Of these 91 EST-SSR primers, 32 random primer pairs were finally selected for validation and genetic variation study in *A. paniculata.* Of these 32 primer pairs, 23 could amplify unambiguous bands, and thirteen showed polymorphic and reproducible bands. **Supplementary Table S5(B)** shows detailed information on 23 primer pairs and their probable gene functions.

#### 3.3.2 SSR analysis

Finally, 13 polymorphic EST-SSR primer pairs were utilized to fingerprint twenty-four accessions of *A.paniculata*. An average of two alleles/primer pairs were spotted, with a total of 26 alleles at 13 marker loci. The percentage polymorphism across all the primer pairs was 100%. Three representing profiles [primer pair ID APSSR 6, APSSR 16 & APSSR 2] are displayed in **Supplementary Image 1(A–C)**. The estimates of various genetic parameters representing the discriminating power of polymorphic SSR primers are shown in **Table 1**. The PIC value varied from 0.149 to 0.465, averaging 0.322 per primer. The resolving power (Rp) ranged from 0.667 to 2.917, with an average value of 1.802/primer. The marker index (MI) and effective marker index (EMI) values also varied from 0.299 to 0.899 and 0.224 to 0.674 per primer, averaging 0.644 and 0.483/primer, respectively.

**Table 1:** Genetic parameters of thirteen polymorphic EST-SSRs used in the study.

S.No.	Primer name	TNB	NPB	PP (%)	PIC	RP	EMR	MI	QM	QND	EMI
1.	APSSR 1	2	2	100	0.326	1.833	2	0.653	1	0.75	0.490
2.	APSSR3	2	2	100	0.345	1.750	2	0.691	1	0.75	0.518
3.	APSSR5	2	2	100	0.283	1.583	2	0.566	1	0.75	0.424
4.	APSSR6	2	2	100	0.450	1.750	2	0.899	1	0.75	0.674
5.	APSSR7	2	2	100	0.149	1.833	2	0.299	1	0.75	0.224
6.	APSSR12	2	2	100	0.465	1.500	2	0.931	1	0.75	0.698
7.	APSSR14	2	2	100	0.299	1.500	2	0.597	1	0.75	0.448
8.	APSSR16	2	2	100	0.352	2.917	2	0.705	1	0.75	0.529
9.	APSSR17	2	2	100	0.387	1.417	2	0.774	1	0.75	0.581
10.	APSSR18	2	2	100	0.247	1.500	2	0.493	1	0.75	0.370
11.	APSSR19	2	2	100	0.247	0.667	2	0.493	1	0.75	0.370
12.	APSSR29	2	2	100	0.274	1.833	2	0.549	1	0.75	0.411
13.	APSSR33	2	2	100	0.361	1.667	2	0.722	1	0.75	0.542
	Average	2	2	100	0.322	1.673	2	0.644	1	0.75	0.483

TNB, NPB, PP, PIC, RP, EMR, MI, QM, QND and EMI refer total number of bands, number of polymorphic bands, percent polymorphism, polymorphic information content, resolving power, effective multiplex ratio, marker index, quality of marker, qualitative nature of data and effective marker index, respectively.

#### 3.3.3. Genetic diversity and relationships study

Genetic diversity was studied using binary data matrices produced by thirteen polymorphic marker loci. The pair-wise genetic similarity coefficient showed 100% genetic similarity among accessions AP4, AP17, and AP18; between AP5 & AP7; among accessions AP8, AP9, AP10 & AP12; between AP6 & AP11, and between AP13 & AP14. However, the minimum genetic similarity (40%) was observed between accession AP1 & AP10 (**Supplementary Table S6**).

We also constructed a UPGMA tree using the corresponding genetic similarity coefficient among the studied accessions (**Supplementary Image 2**). The UPGMA-based dendrogram grouped twenty-four accessions into two distinct clusters, wherein three accessions (AP1, AP2, and AP3) were grouped in Cluster I and twenty-one in Cluster II. Further grouping was observed in cluster II with two sub-clusters- Sub-cluster II (a) and Sub-cluster II (b). Sub-cluster II (a) encompassed three accessions (AP13, AP14, AP22), and cluster II (b) included eighteen accessions (AP4, AP17, AP18, AP23, AP5, AP7, AP8, AP9, AP10, AP12, AP6, AP11, AP15, AP24, AP16, AP19, AP20, and AP21) of different ecotypes.

#### 3.3.4 Analysis of molecular variance (AMOVA)

**Figure 5:**
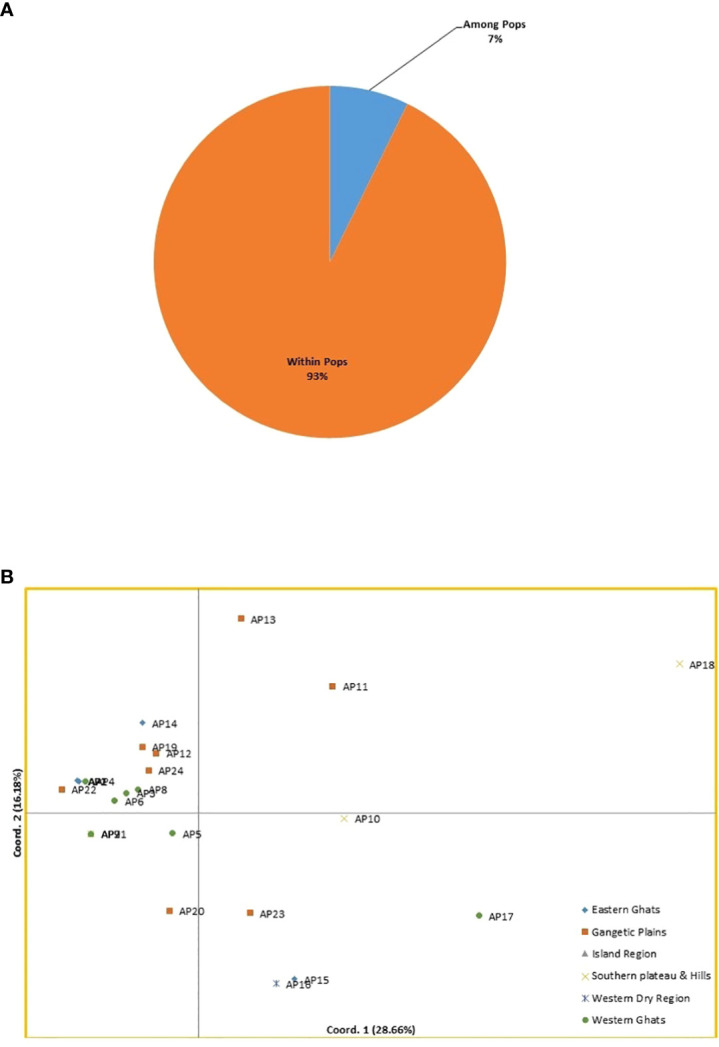
**(A)** Analysis of molecular variance (AMOVA) of twenty-four kalmegh accessions based on EST-SSR primers. **(B)** Two-dimensional distribution of twenty-four kalmegh accessions on PCA biplot.

The AMOVA was used to calculate the variability across and within the *A. paniculata* accessions procured from various agro ecological regions of India. Six populations were considered in the current study depending on agro ecological zones (**Figure 1**). The SSR data showed 7% variation among six populations and 93% variation within a population, as shown in **Figure 5A**. Significant genetic variation was found among and within the accessions of kalmegh (**Supplementary Table S7A**).

#### 3.3.5 Principal coordinate analysis (PCoA)

The PCoA was also computed to get an alternative view of phylogenetic relationships among the twenty-four accessions of *A. paniculata*. The cumulative percentage of variation elucidated by the first three coordinates was 56.05%, with PCo1 contributing 28.66%, PCo2 contributing 16.18%, and PCo3 contributing 11.21%, respectively (**Supplementary Table S7B**). The grouping of accessions on the PCoA biplot was not in accordance with cluster analysis. However, the accession AP18 from Southern Plateau and Hills was distinct and grouped alone on the biplot display (**Figure 5B**).

#### 3.3.6 Population structure-based study

**Figure 6:**
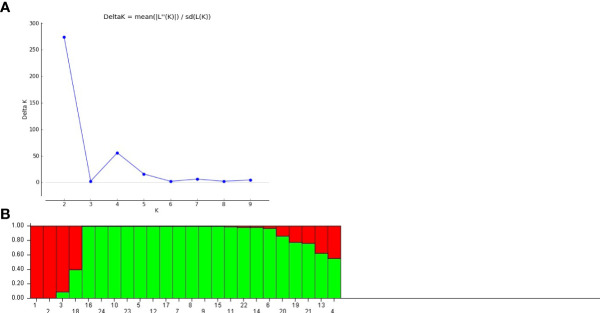
**(A)** Estimation of population using LnP(D) derived delta k value **(B)** Population structure at K= 2 by Evanno table in *A.paniculata* accessions.

Genetic structure of the studied germplasms was also evaluated using Evanno’s method based STRUCTURE software. The total number of genetic populations (k) indicated a clear peak at two with an optimum delta k value, indicating the distribution of two populations across all the studied accessions (**Figure 6A**). As shown in **Figure 6B**, the population I comprised three pure accessions and one admixed accession. However, population II displayed sixteen pure accessions and four admixed accessions. Grouping of studied accessions into different populations was ecotype independent.

### 3.4 Joint analysis of agro-phytochemical and genotypic data

**Figure 7:**
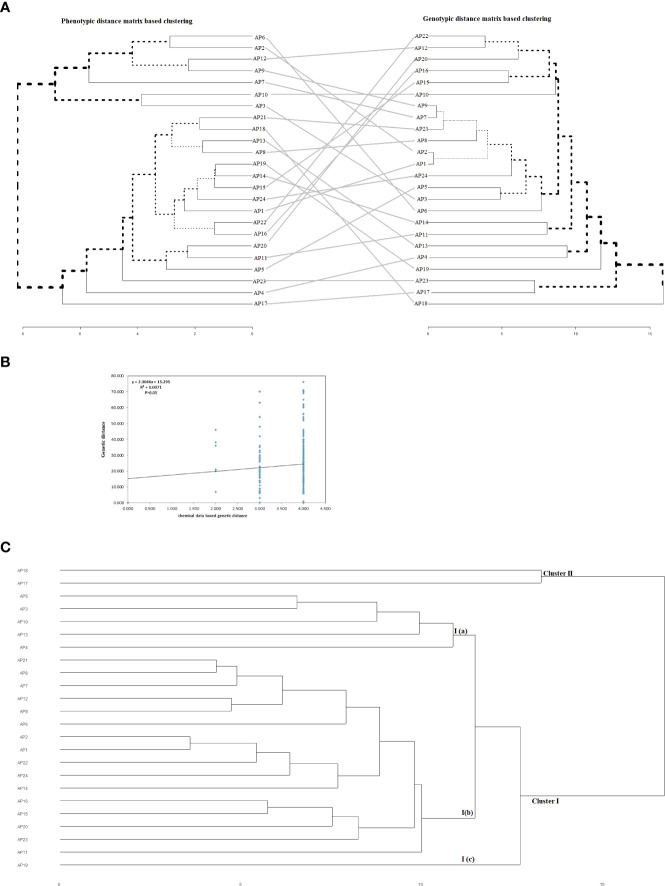
**(A)** Comparative assessment of phenotypic and genotypic data based hierarchical cluster dendrograms. The black lines represent mismatched accessions in-between the two dendrograms from the phenotypic to the genotypic cluster. **(B)** Mantel test representing the relationship between phytochemical and molecular data sets. **(C)** Hierarchical clustering of twenty-four accession of kalmegh using combined agro-phytochemical and molecular datasets.

The phenotypic and genotypic information-based distance matrices were used to generate separate hierarchical clusters that were finally compared with each other. We did not find any individual to be clustered in the same position across the two phylogenetic trees (**Figure 7A**). Also, the Mantel test performed between phytochemical and genotypic datasets showed no significant correlation (R^2 =^ 0.0071, *P*>0.05) besides forming two clear-cut clusters with each dataset (**Figure 7B**). The combined clustering analysis of agro-phytochemical and genotyping data revealed two well-defined clusters (Cluster I and Cluster II) in the current set of materials (**Figure 7C**). A total of twenty-two and two accessions were grouped in Cluster I and ClusterII, respectively. The Cluster I was further subgrouped with five accessions in sub-cluster I(a), sixteen accessions in sub-cluster I(b), and one accession in sub-cluster I(c). No geographical regions or trait-specific grouping of individuals was observed in different clusters and sub-clusters.

## 4 Discussion

*A. paniculata* has gained much attention as the sole plant producing Andrographolide in the last several decades. Commercial cultivars available in kalmegh are ecotype specific and minimal in number to meet global demand. The increased demand for high-quality raw materials warrants the exploration of its genetic potential from wild sources to develop an operative breeding program in kalmegh, following suitable selection techniques. Also, assessing genetic variation present in the indigenous genetic resources may provide the foundation for effective selection response and genetic gain in kalmegh breeding. Moreover, evaluating the genetic mechanism of different indigenous ecotypes of *A. paniculata* are central to its sustainable cultivation. Considering the lack of enough comprehensive information on the genetic diversity in Indian kalmegh ecotypes, the present study exploited both agro-morphological and phytochemical systems to evaluate the genetic diversity in *A. paniculata* ecotypes since the variation in the agro-phytochemical traits in different plant populations may be primarily caused by genetic variation and interaction of environmental conditions. In the present study, specialized metabolic pathway-specific genic SSRs (EST-SSRs) were introduced for the first time to determine the genetic variability among kalmegh accessions and to analyze the inter-relationship between agro-phytochemical traits and EST-SSR markers to speed-up trait-specific breeding in kalmegh.

By analyzing ANOVA for nine agro-morphological traits of studied accessions, we demonstrated that all the accessions have sufficient phenotypic diversity for all traits except leaf length (LL), which could provide the basis of selection in the kalmegh breeding program. A broad range of variation was seen in kalmegh accessions against dry herb yield, plant height, number of secondary branches per plant, days to fifty percent flowering, and andrographolide content, suggesting the diverse genetic makeup of these accessions. These promising accessions from different ecological regions may be valuable resources for augmenting the genetic gains of cultivated varieties (**Supplementary Table S3B**). Similar results were shown by Latto et al. (2008) in *A. paniculata* for morphometric traits.

Various genetic variability parameters were analyzed to determine the value of genetic diversity among experimental sets. The PCV was consistently higher than the GCV, showing the role of environment in the trait expression. However, the difference between PCV and GCV values was estimated to be narrow (<10%) for all traits studied except leaf length (LL), showing significant genetic control and little environmental influence on trait expression (**Supplementary Table S3C**, **Figure 2**). The moderate GCV was observed for leaf width (LW), and the number of secondary branches/plant (NSBP), enhancing the scope of selection due to strict genetic control on the expression of these traits. Heritability estimates predict the breeding value and strengthen the reliability of the phenotypic value of traits in any crop improvement program (Kumar et al., 2014). Simple selection can quickly improve traits with high heritability. Heritability, on the other hand, has proven to be useless without the association of genetic advancement. Heritability estimations and genetic advance (GA) together predict how well selection will work by choosing better genotypes. High heritability with high GA describes the involvement of additive gene action in quantitative traits expression, whereas low GA with high heritability defines the influence of non-additive gene action in the manifestation of quantitative traits. As revealed in **Supplementary Table S3(C)** & **Figure 2**, none of the traits showed high estimates of GA and heritability. However, moderate estimates of GA and high to moderate estimates of heritability were calculated for traits, namely days to fifty percent flowering and dry herb yield, anticipating the efficacy of direct selection in the studied accessions for these traits. Little efforts have been undertaken to understand the role of genetic factors in manifesting quantitative traits in kalmegh. In 2000, Mishra et al. (2000) observed high GCV, heritability, and GA for dry herb yield and plant height in 22 morphologically diverse accessions of kalmegh. Thus, in the present study, dry herb yield and the number of secondary branches per plant could be appropriate selection indexes for parent selection in the hybridization program of kalmegh accessions.

Different multivariate clustering methods were used to classify twenty-four kalmegh accessions based on agro-morphological and phytochemical traits. The silhouette algorithm-based k-mean clustering, Euclidean distance-based clustering, and PCA divided the experimental set into three groups based on agro-morphological attributes (**Figures 3A–C**). Among the nine quantitative traits, the main distinguishing trait that contributed to diversity among different groups in cluster analysis was the proportion of dry herb yield. Overall, the grouping pattern was slightly similar in both heatmap clustering and PCA biplot showing considerable diversity among experimental sets, which might be attributed to gene ontology, soil, and environmental conditions. The hybridization between the accessions with the least genetic similarities could be effective for the production of superior genotypes containing desirable traits. Likewise, all the three clustering methods (k-mean clustering, Euclidean distance-based clustering, and PCA) divided twenty-four accessions into two clear-cut groups based on phytochemical traits (**Figures 4A–C**), demonstrating considerable variance among experimental sets based on chemical concentrations. The distinctive characteristic contributing to diversity among different groups was the share of andrographolide content. Thus, despite growing in the same soil and temperature regime, principal component analysis and cluster analysis allowed the classification of *A.paniculata* accessions into different groups and revealed significant variability in chemical concentrations among experimental sets, which appears to be associated with genetic factors accumulated during selection.

Moreover, our clustering results based on agro-morphological traits and phytochemical traits showed no specific grouping pattern of geographically closer accessions of *A. paniculata*. The propensity to produce such a clustering pattern suggests that geographic segregation might not always dilute the genetic makeup of introductions which leads to diversity in natural populations. In light of this, it seems that accessions’ genetic makeup, as opposed to their eco-geographic origin, significantly influences clustering. This may result from the unrestricted flow of seed from the native place to the area of their domestication. Our results align with the reports of Lattoo et al. (2008) for morphometric traits and Hiremath et al. (2020) for morphometric and chemotypic traits in *A.paniculata.*

Characterization of diversity based on morpho-metric traits may not be reliable as these are vulnerable to ontology and environmental factors. Therefore, it is desirable to assess diversity based on molecular markers and compare them with morphometric traits to attain more realistic results. According to Varshney et al. (2005), molecular markers have become a frequent and crucial tool for assessing genetic relationships and diversity, cultivar identification and development, efficient gene mapping, tagging, and early generation detection of superior genotypes in many crops. In recent years, microsatellites (SSRs) have become a marker of choice due to their high abundance, hyper-variable, reproducible, co-dominance, and discriminatory nature. Simple Sequence Repeats (SSRs) in the transcribed regions are perceived to be more conserved, significant, and transferable across taxonomic borders than the anonymous SSRs (Pashley et al., 2006; Ellis and Burke, 2007). Transcriptome sequencing is nowadays a quick and affordable way to obtain the EST (Expressed sequence tags) sequences required to isolate a massive set of functional SSRs associated with novel genes. Many EST-SSRs have been developed recently in diverse plant species using transcriptome sequences (Wu et al., 2014). In *A. paniculata*, this is the first established report where transcriptome-based EST-SSR markers were developed and validated. Thus, in the present study, we aimed to generate new EST-SSR markers from the already available transcriptome data of kalmegh in our lab through Illumina paired-end RNA-seq technology (published by Garg et al., 2015). The non-redundant combined transcript extracted from the leaf and root transcriptome and annotated to be involved in different metabolic pathways were used for marker development. The markers obtained from these sequences would be more beneficial than genomic sequence data for trait-specific breeding and detection in *A. paniculata*.

In the present study, thirty-two SSRs primer pairs discovered *via* 50 non-redundant transcripts were selected randomly for experimental validation to build a working maker set for kalmegh genetic improvement. Of these 32 primer pairs, over 71.87% (23primer pairs) successfully amplified genomic DNA and over 40% (13primer pairs) produced polymorphic and reproducible bands across twenty-four accessions (**Supplementary Table S5A &B**). Our success rate is comparable to other efforts done in medicinal herbs, where EST-SSRs amplification rate of 60-80%was reported (Gong & Deng, 2010; Sathyanarayana et al., 2017; Vidya et al., 2021). Primer development across intron/exon splice sites, alternate splice sites, or chimeric transcripts could cause marker dropout in genic-SSRs. In the current study, 13 polymorphic EST-SSRs were finally selected to evaluate genetic relationships and diversity among studied accessions. The discriminatory power of these primer pairs was analyzed using different parameters (**Table 1**). The PIC value is assessed by considering both the allelic numbers as well as their frequency distribution across the experimental set. It quantifies the polymorphism for a marker locus (Guo and Elston, 1999). All the kalmegh SSRs validated in the current study showed a moderate PIC value (<0.5), with a mean of 0.322. Reports around the globe suggested that the EST-SSR primers show less polymorphism than genomic SSRs in crop plants due to larger sequence conservation in transcribed regions (Varshney et al., 2005). Our result of PIC was lower than *Coriandrum sativum* (0.38) (Tulsani et al., 2019) *Docynia delavayi* (0.587) (Peng et al., 2021), *Ginkgo biloba* (0.781) (Zhou et al., 2019), and *Quercus petraea* (0.787) (Lupini et al., 2019) identified with EST-SSR markers. The moderate estimates of PIC might be due to the development of SSRs from the metabolic pathway-specific, highly conserved transcribed region of kalmegh. The PIC estimated in our study was comparatively greater than that of the plant species *Rhododendron arboretum* where PIC was reported to be low (0.195) (Sharma et al., 2020). This indicates that the *A. paniculata* loci examined here had a significant level of discernment, reflecting the complexity of genetic diversity and structure (Peng et al., 2021). The Rp, MI and EMI of 13 EST-SSRs were also estimated, indicating the high efficiency of these primers in kalmegh genetic diversity assessment. Varshney et al. (2007) also analyzed the discriminating nature of EST-SSR primers during the evaluation of different species and cultivars of barley (*Hordeum vulgare*). Sahoo et al. (2021) also reported the average MI of 4.84 in Indian Curcuma species using EST-SSR markers which is significantly higher than our study.

The cluster analysis and population structure analysis placed all the twenty-four accession of *A.paniculata* into two groups showing the presence of reasonable variability among them that could be potential sources for selecting parents for breeding purposes. In UPGMA-based clustering (**Supplementary Image 2**), thirteen accessions of five different sub-groups were found to be genetically identical, showing the inability of EST-SSR primers to differentiate them at the genetic level. This might be due to limited sampling and similar topography of the regions represented by these accessions, as seven of these thirteen accessions belong to the western regions of the country. However, this could also be due to the low genetic variability present among accessions of represented regions (Shiferaw et al., 2012). Bayesian model-based population structuring considered individuals with a probability score of >0.80 as genetically pure and a score of<0.80 as admixed type (**Figure 6B**). Mixing of pure individuals with few admixed accessions was observed in both the populations derived from model-based study, which could be due to the breeding behavior of the studied plant (Kumar et al., 2020). AMOVA and PCoA also explained substantial genetic diversity among the studied accessions of six agroecologically grouped populations. However, ANOVA based genetic differentiation showed maximum variation within agroecological regions rather than between agroecological regions indicating frequent gene flow through seed or out-crossing across different agroecological populations (Tiwari et al., 2016) (**Figure 5A**). Positioning of studied accessions on PCoA biplot was not in congruence with Cluster and STRUCTURE analysis (**Figure 5B**). The accession AP18 from Southern Plateau and Hills was very distinct on the biplot otherwise no specific grouping pattern was observed in PCoA analysis. Overall, different clustering methods used in the present study could not able to classify studied accession with their geographical distribution. The low genetic differentiation among different (six) agroecological populations could be interpreted as genetic drift due to seed dispersal, human intervention, or cross-pollination. Seed dispersal or seed exchange may result in an increase in allelic diversity among diverse populations regardless of their geographical isolation causing enhanced genetic diversity in local germplasm (Louette et al., 1997). Also, the reproductive biology of the plant might have contributed to the distribution of alleles across the regionally isolated population. Although the anthecology of *A. paniculata* favors self-pollination, there are records of substantial outcrossing (around 4%) through insect-pollination. (Shiferaw et al., 2012; Tiwari et al., 2016). Poor sampling size of different agroecological populations could be another reason for the low genetic diversity among different populations. Thus, more detailed studies with larger sampling sizes from extended geographical regions could draw a concrete inference (Sahoo et al., 2021). Previously, evaluation of genetic diversity among the geographically isolated germplasm of *A.paniculata* was carried out using various dominant molecular markers (Lattoo et al., 2008; Minz et al., 2013; Tiwari et al., 2016; Kumar et al., 2020; Hiremath et al., 2020) the outcomes of which are in line with our results. However, in the present study, metabolic pathway specific EST-SSR markers were designed and used to determine the genetic diversity among twenty-four ecotypes of *A. paniculata*. These noval SSR loci displayed relatively high polymorphism levels and could be an important tool for investigating genetic diversity and assessing effective strategies for selective breeding and conservation in *A. paniculata.*

Further, the inconsistency observed between hierarchical clusters identified by phenotypic and genotypic distance matrices could be due to the negligible correlation observed between them and enormous genotype x environment (GxE) interaction effects observed for quantitatively inherited agro-phytochemical traits (**Figure 7A**). This observation was also supported by the Mantel test drawn between the phytochemical and genotypic distance matrix (**Figure 7B**). The negligible correlation could also be due to the non-adaptive nature of variation created by EST-SSR markers, unlike quantitative agronomic or phytochemical traits (Singh et al., 1991). Similar results showing the discrepancy between phenotypic and genotypic datasets were reported by several workers in different crops (Hartings et al., 2008; Soriano et al., 2016; Agre et al., 2019; Darkwa et al., 2020). Therefore, an approach using combined datasets of genotypic and phenotypic information to capture entire genetic variability present in the plant populations and assess genetic diversity was suggested by Alves et al. (2013) and da Silva et al. (2017). The joint cluster analysis performed with agro-phytochemical and genotypic datasets generated two genetic groups in the experimental sets of kalmegh with regrouping of individuals in different clusters, unlike separate hierarchical clustering computed by different datasets in the present study (**Figure 7C**). The genetic diversity assessed by joint cluster analysis could have significant implications for *A. paniculata* genetic improvement. The genetic groups identified in different clusters could be better utilized as trait progenitors in the different selection and hybridization programs, thereby enlarging the genetic base of the kalmegh breeding program and maximizing genetic gain. Our results are in line with the finding of Agre et al. (2019); Darkwa et al. (2020), and Alves et al. (2013), who unlocked genetic diversity in their studies using phenotypic, molecular, and combined datasets.

## 5 Conclusion

Adequate genetic diversity is crucial to project appropriate breeding programs and develop improved varieties in kalmegh genetic improvement. In the present study, genic EST-SSR markers along with agronomic and phytochemical traits, were used to assess genetic diversity among twenty-four kalmegh accessions collected from diverse agroecological zones of India. Our results on genetic diversity revealed sufficient genetic variation in the studied population, which can be exploited for kalmegh genetic improvement and germplasm conservation. The agro-morphological descriptors, days to fifty percent flowering and dry herb yield identified as potential selection index in the present study could be utilized further in kalmegh genetic improvement program. The low genetic differentiation observed among the different agroecological populations could be improved by increasing the sample size from extended geographical regions. In our study, the inconsistency observed between genotypic and phenotypic information could be resolved by enhancing genome-wide information with more number of functional EST-SSR markers to obtain concrete outcomes from them. Our results on combined datasets expanded the scope of selective breeding in kalmegh by utilizing different trait-specific parental lines grouped in different genetic clusters generated by phenotypic and genotypic information. However, further research on economic traits using more genetic and genomic resources can complement the current study and generate more reliable information on Indian kalmegh ecotypes.

## Data availability statement

The original contributions presented in the study are included in the article/**Supplementary Material**. Further inquiries can be directed to the corresponding author.

## Author contributions

GT: Conceived and designed the whole research. Designed EST-SSR markers and performed all statistical analyses. Wrote the manuscript; TC: Collected the phenotype data and performed molecular genotyping; AG: Provided experimental materials from the gene bank; KS: Performed chemical analysis; BK: Supported in lab activity required to perform whole research. All authors contributed to the article and approved the submitted version.

## Acknowledgments

We are grateful to the Late Dr. Hari Om Misra, Ex-Senior Principal Scientist, Division of Plant Breeding and Genetic Resource Conservation, Council of Scientific and Industrial Research-Central Institute of Medicinal and Aromatic Plants (CIMAP), Lucknow who collected the kalmegh germplasm from different parts of India. The authors are also thankful to Dr. Sumit Ghosh, Senior Principal Scientist, Division of Plant Biotechnology, CSIR-CIMAP for providing combined transcript sequences of AP variety ‘CIM-Megha’ and giving guidance for SSR marker development. The institutional communication number of the publication is CIMAP/PUB/2022/99.

## Conflict of interest

The authors declare that the research was conducted in the absence of any commercial or financial relationships that could be construed as a potential conflict of interest.

## Publisher’s note

All claims expressed in this article are solely those of the authors and do not necessarily represent those of their affiliated organizations, or those of the publisher, the editors and the reviewers. Any product that may be evaluated in this article, or claim that may be made by its manufacturer, is not guaranteed or endorsed by the publisher.
