# Supplementary material for: Maximizing genetic gain through unlocking genetic variation in different ecotypes of kalmegh (Andrographis paniculata (Burm. f.) Nee)
Source: Front Plant Sci. 2022 Nov 7;13:1042222. doi: 10.3389/fpls.2022.1042222 (PMC9677111; doi:10.3389/fpls.2022.1042222)
Supplement: Supplementary file 1 [file DataSheet_1.docx]

**Supplementary Table S1:** List of twenty-four accessions of *A. paniculata* collected from different ecotypes of India

**Supplementary Table S2**: Protocol for plant DNA isolation given by Khanuja et al. (1998)

**Supplementary Table S3(A):** Analysis of variance for nine agro-morphological traits in twenty-four accessions of *A.paniculata*

**Supplementary Table S3(B):** Mean performance of twenty-four *A.paniculata* accessions for nine agro-morphological traits pooled over two consecutive seasons

**Supplementary Table S3(C):** Estimates of genetic variability parameters for nine agro-morphological traits in *A. paniculata*

**Supplementary Table S4(A):** Eigenvalue, explained and cumulative variances in the principal component analysis (PCA) used to classify *A. paniculata* genotypes based on agro-morphological data

**Supplementary Table S4(B):** Mean of the phytochemical traits of twenty-four accessions of *A.paniculata* estimated and pooled over two consecutive years.

**Supplementary Table S4C):** Eigenvalue, explained, and cumulative variances in the principal component analysis (PCA) based on phytochemical data.

**Supplementary Table S5(A):** Characterization of specialized metabolic pathway-specific EST-SSRs in *A.paniculata*

**Supplementary Table S5(B):** List of 23 pairs of amplified EST-SSR primers and their annotation

**Supplementary Image1:** SSR amplification profile of primer pairs APSSR6 **(A)**, 16 **(B)**, and 21 **(C)**. Lane M: DNA molecular standards with length (bp) on left. Lane1-24: genotypes of 24 kalmegh accessions (AP1-AP24) as mentioned in S1.

**Supplementary Table S6:** Jaccard’s similarity coefficient measured among twenty-four accessions of Kalmegh (*Andrographis paniculata*)

**Supplementary Image 2:** UPGMA-based dendrogram of twenty-four accessions of kalmegh using thirteen EST-SSR markers

**Supplementary Table S7:** **(A)** Summary statistics of AMOVA **(B)** Percentage of variation explained by first three axes in PCoA using EST-SSR markers
