# Supplementary material for: Maximizing genetic gain through unlocking genetic variation in different ecotypes of kalmegh (Andrographis paniculata (Burm. f.) Nee)
Source: Front Plant Sci. 2022 Nov 7;13:1042222. doi: 10.3389/fpls.2022.1042222 (PMC9677111; doi:10.3389/fpls.2022.1042222)
Supplement: Supplementary file 4 [file Table_1.docx]

**Supplementary Table S1:** List of twenty-four accessions of *A. paniculata* collected from different ecotypes of India

| **Agro-ecological Regions** | **Location** | **Accession code** | **Numbers** | **Longitude** | **Latitude** | **Altitude (m)** |
| --- | --- | --- | --- | --- | --- | --- |
| **Gangetic Plains** | Barauni | AP19 | 1 | 85.9914 | 25.4657 | 49 |
| **Gangetic Plains** | Sultanpur | AP20 | 1 | 82.066 | 26.2585 | 95 |
| **Gangetic Plains** | Jaunpur | AP22, AP23 | 2 | 82.6837 | 25.746 | 63 |
| **Gangetic Plains** | Lucknow | AP24 | 1 | 80.9462 | 26.8467 | 123 |
| **Gangetic Plains** | Agra | AP11, AP12, AP13 | 3 | 78.0080 | 27.1767 | 171 |
| **Eastern Ghats** | Madhurai | AP1, AP2 | 2 | 78.1198 | 9.9252 | 101 |
| **Eastern Ghats** | Ranchi | AP14, AP15 | 2 | 85.3096 | 23.3441 | 700 |
| **Western Ghats** | Ahemdabad | AP3, AP4, AP5, AP6 | 4 | 72.5714 | 23.0225 | 53 |
| **Western Ghats** | Nagpur | AP7, AP8 | 2 | 79.0882 | 21.1458 | 295 |
| **Western Ghats** | Gujarat | AP17 | 1 | 71.1924 | 22.2587 | 293 |
| **Western Dry region** | Jaipur | AP16 | 1 | 75.7873 | 26.9124 | 1417 |
| **Southern plateau & Hills** | Andhra Pradesh | AP10, AP18, AP21 | 3 | 79.74 | 15.9129 | 1000 |
| **Island Region** | Portblair | AP9 | 1 | 92.7265 | 11.6234 | 79 |
