# Supplementary material for: Maximizing genetic gain through unlocking genetic variation in different ecotypes of kalmegh (Andrographis paniculata (Burm. f.) Nee)
Source: Front Plant Sci. 2022 Nov 7;13:1042222. doi: 10.3389/fpls.2022.1042222 (PMC9677111; doi:10.3389/fpls.2022.1042222)
Supplement: Supplementary file 5 [file Table_2.docx]

Supplementary Table S2: Protocol for plant DNA isolation given by Khanuja et al. (1998)

Steps:

1. Grind the plant material in liquid nitrogen (1 gm fresh tissue or 0.2-0.5 g of dry tissue).
2. Transfer the material to 2ml tube and add 1 ml of freshly prepared extraction buffer, mix by inversion to a slurry.
3. Incubate at 60 ◦C in a shaking water bath for 1–2 h (dry samples may require overnight incubation at 37 ◦C).
4. Add equal volume (1ml) of chloroform: isoamyl alcohol (24:1) and mix by inversion for about 15 min.
5. Spin at 8000 rpm for 10 min at 25–30 ◦C.
6. Carefully transfer the upper clear aqueous layer to another 2ml polypropylene tube.
7. Add 250 ul of 5M NaCl and mix properly (do not vortex).
8. Add 0.6 volume of isopropanol and let the mixture stand at room temperature for 1 h.
9. After mixing with isopropanol, sample stored at 4^o^C overnight after the samples can be centrifuged at 10,000 rpm for 10 min at 25–30 ◦C.
10. Discard the supernatant and wash the pellet with 80% ethanol.
11. Dry the pellet in a vacuum for 15 min and dissolve it in 0.3 ml of high salt TE buffer.
12. Add 1.5 µl of RNase A and incubate at 37 ◦C for 30 min.
13. Extract with equal volume of chloroform: isoamyl alcohol (24:1).
14. Transfer the aqueous layer to a fresh 1.5 ml microfuge tube and add 2 volumes of cold ethanol.
15. Spin at 10,000 rpm for 10 min at 25–30 ◦C.
16. Wash the pellet with 80% ethanol.
17. Dry the pellet in a vacuum and dissolve in 200 µl of sterile double distilled water.
18. DNA concentrations can be measured by running aliquots on an 0.8% agarose gel or by taking the absorbance at 260 nm.

Reagents and chemicals required

• Tris-Cl pH 8.0 (1.0 M); EDTA pH 8.0 (0.5 M); NaCl (5.0 M); CTAB (20%); Chloroform: Isoamyl alcohol (24:1 v/v); Polyvinylpyrrolidone; and β-mercaptoethanol.

• Extraction buffer: 100 mM Tris-Cl (pH 8.0), 25 mM EDTA, 1.5 M NaCl, 2.5% CTAB, 0.2% β-mercaptoethanol (v/v) (added immediately before use) and 1% PVP (w/v) (added immediately before use).

• High salt TE buffer: 1 M NaCl, 10 mM Tris-Cl (pH 8.0) and 1 mM EDTA
