# Supplementary material for: Maximizing genetic gain through unlocking genetic variation in different ecotypes of kalmegh (Andrographis paniculata (Burm. f.) Nee)
Source: Front Plant Sci. 2022 Nov 7;13:1042222. doi: 10.3389/fpls.2022.1042222 (PMC9677111; doi:10.3389/fpls.2022.1042222)
Supplement: Supplementary file 6 [file Table_3.docx]

| Characters | Days to 50% flowering (DFF) | Days to maturity  (DM) | Plant height  (PH) | Number of nodes per plant (NNP) | Number of secondary branches per plant (NSBP) | Leaf length  (LL) | Leaf width  (LW) | Inflorescence length  (IL) | Dry herb yield  (DHY) |
| --- | --- | --- | --- | --- | --- | --- | --- | --- | --- |
| Replication  (df=2) | 56.88*** | 83.53*** | 7.96 | 1.98 | 14.88 | 4.15 | 0.010 | 0.08 | 26.32 |
| Genotypes  (df=23) | 122.79*** | 40.11*** | 43.76*** | 9.74*** | 27.58*** | 5.51 | 0.41** | 0.41*** | 248.48*** |
| Error  (df=46) | 5.54 | 4.29 | 8.70 | 3.01 | 5.73 | 4.27 | 0.14 | 0.13 | 65.12 |

**Supplementary Table S3(A):** Analysis of variance for nine agro-morphological traits in twenty-four accessions of *A.paniculata*

***-P<0.05, **-P<0.01, and ***- P<0.001**

**Supplementary Table S3(B):** Mean performance of twenty-four *A.paniculata* accessions for nine agro-morphological traits pooled over two consecutive seasons

| Genotypes | Days to 50% flowering (DFF) | Days to maturity  (DM) | Plant height  (PH) | Number of nodes per plant (NNP) | Number of secondary branches per plant (NSBP) | Leaf length  (LL) | Leaf width  (LW) | Inflorescence length  (IL) | Dry herb yield  (DHY) |
| --- | --- | --- | --- | --- | --- | --- | --- | --- | --- |
| AP1 | 127 | 202 | 66.3 | 21 | 16 | 5.6 | 1.9 | 3.7 | 94.7 |
| AP2 | 121 | 195 | 62.5 | 19 | 16 | 6.2 | 2.1 | 3.4 | 75.3 |
| AP3 | 128 | 201 | 63.4 | 23 | 24 | 4.4 | 3.1 | 4.6 | 100 |
| AP4 | 131 | 204 | 68.7 | 20 | 19 | 12.6 | 1.9 | 4.1 | 88 |
| AP5 | 133 | 203 | 65.7 | 21 | 21 | 7 | 1.9 | 4.5 | 102 |
| AP6 | 129 | 203 | 62.1 | 20 | 16 | 6.7 | 1.8 | 3.7 | 74.2 |
| AP7 | 115 | 192 | 59 | 19 | 18 | 6.7 | 1.8 | 4.4 | 107.6 |
| AP8 | 125 | 193 | 65.2 | 21 | 21 | 6.7 | 1.8 | 3.8 | 91.2 |
| AP9 | 131 | 199 | 56.8 | 18 | 14 | 6.2 | 2.2 | 3.1 | 100.6 |
| AP10 | 134 | 206 | 64.1 | 21 | 20 | 6.6 | 2.9 | 3.7 | 95.5 |
| AP11 | 127 | 200 | 67.5 | 24 | 22 | 7 | 1.8 | 4 | 89.5 |
| AP12 | 132 | 196 | 57.8 | 20 | 16 | 6.4 | 1.9 | 3.5 | 94.4 |
| AP13 | 129 | 196 | 64.3 | 21 | 20 | 6.7 | 1.8 | 3.8 | 103.3 |
| AP14 | 132 | 198 | 67.1 | 21 | 16 | 6.6 | 1.7 | 3.8 | 87.3 |
| AP15 | 131 | 197 | 67.9 | 22 | 19 | 6.9 | 1.8 | 3.8 | 90.8 |
| AP16 | 131 | 197 | 68.3 | 23 | 16 | 6.5 | 1.8 | 3.8 | 79.2 |
| AP17 | 143 | 203 | 68.8 | 24 | 10 | 6.1 | 1.8 | 4 | 71.9 |
| AP18 | 134 | 195 | 66.2 | 21 | 19 | 7.1 | 1.7 | 4.4 | 88.4 |
| AP19 | 135 | 198 | 65.8 | 21 | 19 | 6.8 | 1.7 | 3.6 | 87.1 |
| AP20 | 121 | 197 | 63.3 | 22 | 22 | 6.5 | 1.7 | 4.3 | 92.2 |
| AP21 | 132 | 196 | 71.5 | 20 | 19 | 6.8 | 1.6 | 4.3 | 96.2 |
| AP22 | 128 | 195 | 69 | 22 | 15 | 6.6 | 1.7 | 3.9 | 86.2 |
| AP23 | 115 | 200 | 71.5 | 25 | 19 | 7.1 | 2 | 4 | 93.2 |
| AP24 | 135 | 200 | 66.2 | 23 | 19 | 6.8 | 1.9 | 3.6 | 90.6 |
| Grand mean | 129.15 | 198.49 | 65.37 | 21.35 | 18.11 | 6.78 | 1.93 | 3.90 | 90.81 |
| SEm | 1.36 | 1.19 | 1.70 | 1.00 | 1.38 | 1.19 | 0.22 | 0.21 | 4.66 |
| Range | 115-143 | 192-206 | 56.8-75.92 | 18-25 | 10-24 | 4.4-12.6 | 1.6-3.1 | 3.1-4.6 | 71.9-107.6 |
| CD (5%) | 3.87 | 3.40 | 4.84 | 2.85 | 3.94 | 3.39NS | 0.62 | 0.59 | 13.26 |
| CD (1%) | 5.16 | 4.54 | 6.47 | 3.81 | 5.25 | 4.53NS | 0.83 | 0.79 | 17.70 |

Where, NS= non-significant

**Supplementary Table S3(C):** Estimates of genetic variability parameters for nine agro-morphological traits in *A. paniculata*

| Characters | Vg | Vp | PCV(%) | GCV(%) | h^2^ | GA |
| --- | --- | --- | --- | --- | --- | --- |
| DFF | 39.08 | 44.62 | 5.17 | 4.84 | 87.59 | 12.05 |
| DM | 11.94 | 16.23 | 2.03 | 1.74 | 73.58 | 6.10 |
| PH | 11.69 | 20.39 | 6.91 | 5.23 | 57.33 | 5.33 |
| NNP | 2.24 | 5.26 | 10.74 | 7.01 | 42.67 | 2.01 |
| NSBP | 7.28 | 13.02 | 19.92 | 14.90 | 55.94 | 4.16 |
| LL | 0.41 | 4.68 | 31.92 | 9.50 | 8.86 | 0.39 |
| LW | 0.09 | 0.23 | 24.94 | 15.33 | 37.79 | 0.37 |
| IL | 0.09 | 0.22 | 12.18 | 7.87 | 41.80 | 0.41 |
| DHY | 126.24 | 61.12 | 12.37 | 8.61 | 48.41 | 11.20 |

Where, Vg= Genotypic variance; Vp= Phenotypic variance; PCV= Phenotypic coefficient of variation; GCV= Genotypic coefficient of variation; h^2^_=_ heritability; GA= Genetic advance
