# Supplementary material for: Maximizing genetic gain through unlocking genetic variation in different ecotypes of kalmegh (Andrographis paniculata (Burm. f.) Nee)
Source: Front Plant Sci. 2022 Nov 7;13:1042222. doi: 10.3389/fpls.2022.1042222 (PMC9677111; doi:10.3389/fpls.2022.1042222)
Supplement: Supplementary file 7 [file Table_4.docx]

**Supplementary Table S4(A):** Eigenvalue, explained and cumulative variances in the principal component analysis (PCA) used to classify *A. paniculata* genotypes based on agro-morphological data

| Principal Component | Eigenvalue | Variance(%) | Cumulative variance (%) |
| --- | --- | --- | --- |
| PC1 | 2.25 | 25.03 | 25.03 |
| PC2 | 2.10 | 23.37 | 48.40 |
| PC3 | 1.59 | 17.66 | 66.06 |
| PC4 | 1.12 | 12.45 | 78.51 |
| PC5 | 0.68 | 7.58 | 86.09 |
| PC6 | 0.46 | 5.11 | 91.20 |
| PC7 | 0.35 | 3.91 | 95.11 |
| PC8 | 0.24 | 2.65 | 97.76 |
| PC9 | 0.20 | 2.24 | 100.00 |

**Supplementary Table S4(B):** Mean of the phytochemical traits of twenty-four accessions of *A.paniculata* estimated and pooled over two consecutive years.

| Accession code | Andrographolide (AG) | Neoandrographolide  (NAG) | 14-deoxy-11,12-didehydro-andrographolide (DDAG) | Andrographanin (AN) |
| --- | --- | --- | --- | --- |
| AP1 | 3.74 | 1.01 | 0.06 | 0 |
| AP2 | 3.45 | 1.11 | 0.59 | 0.02 |
| AP3 | 4 | 0.68 | 0.05 | 0.02 |
| AP4 | 3.23 | 0.43 | 0.11 | 0.00 |
| AP5 | 3.15 | 0.73 | 0.09 | 0.00 |
| AP6 | 3.17 | 0.67 | 0.06 | 0.03 |
| AP7 | 4.07 | 0.93 | 0.06 | 0.05 |
| AP8 | 2.17 | 0.75 | 0.06 | 0.00 |
| AP9 | 2.18 | 0.64 | 0.14 | 0.03 |
| AP10 | 3.42 | 0.95 | 0.08 | 0.04 |
| AP11 | 4.17 | 1.01 | 0.15 | 0.16 |
| AP12 | 4.28 | 1.14 | 0.2 | 0.00 |
| AP13 | 2.4 | 0.4 | 0.08 | 0.01 |
| AP14 | 2.33 | 0.62 | 0.04 | 0.01 |
| AP15 | 2.63 | 0.65 | 0.07 | 0.06 |
| AP16 | 3.64 | 0.68 | 0.11 | 0.06 |
| AP17 | 3.73 | 0.37 | 0.07 | 0.01 |
| AP18 | 4 | 0.47 | 0.1 | 0.03 |
| AP19 | 2.84 | 0.64 | 0.08 | 0.01 |
| AP20 | 2.4 | 0.31 | 0.06 | 0.00 |
| AP21 | 3.89 | 1.22 | 0.08 | 0.03 |
| AP22 | 2.74 | 0.31 | 0.07 | 0.01 |
| AP23 | 1.21 | 0.4 | 0.26 | 0.01 |
| AP24 | 3.18 | 0.54 | 0.06 | 0.01 |

| Principal Component | Eigenvalue | Variance(%) | Cumulative variance (%) |
| --- | --- | --- | --- |
| PC1 | 1.87 | 46.85 | 46.85 |
| PC2 | 1.05 | 26.33 | 73.18 |
| PC3 | 0.71 | 17.89 | 91.07 |
| PC4 | 0.36 | 8.93 | 100.00 |

**Supplementary Table S4(C):** Eigenvalue, explained and cumulative variances in the principal component analysis (PCA) based on phytochemical data.
