# Supplementary material for: Maximizing genetic gain through unlocking genetic variation in different ecotypes of kalmegh (Andrographis paniculata (Burm. f.) Nee)
Source: Front Plant Sci. 2022 Nov 7;13:1042222. doi: 10.3389/fpls.2022.1042222 (PMC9677111; doi:10.3389/fpls.2022.1042222)
Supplement: Supplementary file 8 [file Table_5.docx]

**Supplementary Table S5(A):** Characterization of specialized metabolic pathway-specific EST-SSRs in *A.paniculata*

| Parameters | Values |
| --- | --- |
| Total number of ESTs searched | 50 |
| Total size of examined sequences | 76,636bp |
| Total number of ESTs with SSRs | 50 |
| Total number of ESTs with single SSR | 27 |
| Total number of ESTs with more than one SSR | 23 |

**Supplementary Table S5(B):** List of 23 pairs of amplified EST-SSR primers and their annotation

| S.No. | Marker name | Primer sequence5’-3’ | Expected size | Tm(^0^C) | Blastx annotation |
| --- | --- | --- | --- | --- | --- |
| 1. | APSSR1 | F:AATTTAGGGCTAGCTTGGAG | 131 | 50.5 | UDP-glycosyltransferase |
|  |  | R:GACTCGAACCTAATCATTGTG |  |  |  |
| 2. | APSSR2 | F:GGTCATTGTAATGAAGAGACG | 136 | 50.9 | Terpene synthase20; UDP-glycosyltransferase |
|  |  | R:CATTTCTCCAGACACACTTTC |  |  |  |
| 3. | APSSR3 | F:TCCTCAACGGTCACTAAAAC | 150 | 54.7 | UDP-glycosyltransferase22 |
|  |  | R:GGTGGTGCAGAATTGTATG |  |  |  |
| 4. | APSSR4 | F:GCACAGATATTGTGACTGGAG | 154 | 56.9 | 1-hydroxy-2-methyl-2-(E)-butenyl-4-diphosphate synthase |
|  |  | R:GATGTCTGATTCTAAGCCTGA |  |  |  |
| 5. | APSSR5 | F:CCTTCATTGCTACACATTCTC | 149 | 56.8 | Terpene synthase 13 |
|  |  | R:ATGTCTGGAACTGAGGGTTAG |  |  |  |
| 6. | APSSR6 | F:AAGCATCAAACACACTCTCAT | 140 | 53.9 | NADPH-cytochome P450 reductase (CPR3); terpene synthase 29 (TPS29); NADPH-cytochrome P450 reductase (CPR1) |
|  |  | R:GGATAAGATCATTTGGGATGT |  |  |  |
| 7. | APSSR7 | F:GATTAACCCTGGTGAAGACTC | 189 | 54.9 | NADPH-cytochrome P450 reductase (CPR2) |
|  |  | R:ATGGGAAATGAGAGTAACCAT |  |  |  |
| 8. | APSSR11 | F:GGAGCTTAAGACTGTTTGGTT | 152 | 57.3 | 1-deoxy-D-xylulose-5-phosphate synthase (DXS1); Geranylgeranyl pyrophosphate synthase 4 |
|  |  | R:CTACCCCCTTCCTCTCTCT |  |  |  |
| 9. | APSSR12 | F:TACACAACACATCACCAGCTA | 138 | 55.9 | Terpene synthase 19 (TPS19);  NADPH-cytochrome P450 reductase (CPR3) gene |
|  |  | R:GAACTGCTTCTGCAATAAGTG |  |  |  |
| 10. | APSSR14 | F:GCGAGAACACATGCTAAAGTA | 152 | 55.5 | Kaurene synthase-like 2 (KSL2);  kaurene synthase 1 (KS1);  NADPH-cytochrome P450 reductase (CPR4) gene |
|  |  | R:CCTTCTCCACATTCATCACT |  |  |  |
| 11. | APSSR15 | F:AGGTTGTAGGAGGAGCAGTAG | 154 | 57.8 | Oxidosqualene cyclase 10 (OSC10);  NADPH-cytochrome P450 reductase (CPR1) gene |
|  |  | R:CACCCATTCCTATTACACTTG |  |  |  |
| 12. | APSSR16 | F:GAGTAGCAACTAGCAAAGCAG | 153 | 56.8 | ent-copalyl diphosphate synthase (CDPS2) gene |
|  |  | R:AAGAAGTTCCAGGAGAGAAGA |  |  |  |
| 13. | APSSR17 | F:AATCCTTCTGGTAGCTTAACG | 151 | 55.9 | UDP-glycosyltransferase 9 |
|  |  | R:AAGTTCACAAAGGAAGAGGTC |  |  |  |
| 14. | APSSR18 | F:TTCTGCTTCTGCTTCTGTACT | 149 | 54.9 | 1-deoxy-D-xylulose-5-phosphate synthase;  UDP-glycosyltransferase 14;  UDP-glycosyltransferase |
|  |  | R:TCTTCTTCTTCTTCGCTATCA |  |  |  |
| 15. | APSSR19 | F:AGAAGAGAAAGGGGAAGAATC | 196 | 55.9 | terpene synthase 32 (TPS32);  UDP-glycosyltransferase 4;  oxidosqualene cyclase 8 (OSC8) |
|  |  | R:CGTTAAATAGAGGGAGGGATA |  |  |  |
| 16. | APSSR21 | F:TGTTCCGAATATGAAGCTCT | 175 | 54.5 | UDP-glycosyltransferase;  Terpene synthase 25 (TPS25) |
|  |  | R:GATTCTTCCCCTTTCTCTTCT |  |  |  |
| 17. | APSSR24 | F:GAGCAACAAACAAACTCACTT | 152 | 55.3 | NADPH-cytochrome P450 reductase (CPR3); ent-copalyl diphosphate synthase (CDPS1) |
|  |  | R:AGAGTTGGGGTCTTGACAG |  |  |  |
| 18. | APSSR25 | F:GGTTGATCGATATGATAGTTCC | 150 | 57.2 | UDP-glycosyltransferase 8;  Terpene synthase 37 (TPS37**)** |
|  |  | R:ATAAGGATACAGACCGTCCTC |  |  |  |
| 19. | APSSR28 | F:CTAAGGCTATACACGAGGTCA | 145 | 56.8 | UDP-glycosyltransferase 6 |
|  |  | R:GGAAGCTATCATCTCCTGATT |  |  |  |
| 20. | APSSR29 | F:CTTCTTCTCCTTTCCACTAGG | 148 | 56.8 | UDP-glycosyltransferase 19 |
|  |  | R:CATAGGGCATGATACTTTCTG |  |  |  |
| 21. | APSSR30 | F:GATAGTCTGTGTTTCGAGTGG | 146 | 55.9 | terpene synthase 39 (TPS39);  terpene synthase 22 (TPS22) |
|  |  | R:AGATATGGTGTTCGAGGACTT |  |  |  |
| 22. | APSSR32 | F:AGAGCAAAAGGTCCAAAATAC | 169 | 53.9 | NADPH-cytochrome P450 reductase (CPR2)  terpene synthase 24 (TPS24) |
|  |  | R:AGATGATATCGATGGATTACG |  |  |  |
| 23. | APSSR33 | F:GATTTCTTGATGAGCTCCAG | 151 | 55.9 | terpene synthase 21 (TPS21)  terpene synthase 9 (TPS9) |
|  |  | R:GCTCGAACGTGGTAAGAAG |  |  |  |
