# Supplementary material for: Maximizing genetic gain through unlocking genetic variation in different ecotypes of kalmegh (Andrographis paniculata (Burm. f.) Nee)
Source: Front Plant Sci. 2022 Nov 7;13:1042222. doi: 10.3389/fpls.2022.1042222 (PMC9677111; doi:10.3389/fpls.2022.1042222)
Supplement: Supplementary file 9 [file Table_6.docx]

| Genotypes | AP1 | AP2 | AP3 | AP4 | AP5 | AP6 | AP7 | AP8 | AP9 | AP10 | AP11 | AP12 | AP13 | AP14 | AP15 | AP16 | AP17 | AP18 | AP19 | AP20 | AP21 | AP22 | AP23 | AP24 |
| --- | --- | --- | --- | --- | --- | --- | --- | --- | --- | --- | --- | --- | --- | --- | --- | --- | --- | --- | --- | --- | --- | --- | --- | --- |
| AP1 | 1 |  |  |  |  |  |  |  |  |  |  |  |  |  |  |  |  |  |  |  |  |  |  |  |
| AP2 | 0.94 | 1.00 |  |  |  |  |  |  |  |  |  |  |  |  |  |  |  |  |  |  |  |  |  |  |
| AP3 | 0.87 | 0.93 | 1.00 |  |  |  |  |  |  |  |  |  |  |  |  |  |  |  |  |  |  |  |  |  |
| AP4 | 0.68 | 0.74 | 0.79 | 1.00 |  |  |  |  |  |  |  |  |  |  |  |  |  |  |  |  |  |  |  |  |
| AP5 | 0.55 | 0.63 | 0.70 | 0.92 | 1.00 |  |  |  |  |  |  |  |  |  |  |  |  |  |  |  |  |  |  |  |
| AP6 | 0.58 | 0.64 | 0.74 | 0.78 | 0.88 | 1.00 |  |  |  |  |  |  |  |  |  |  |  |  |  |  |  |  |  |  |
| AP7 | 0.57 | 0.64 | 0.72 | 0.87 | 1.00 | 0.90 | 1.00 |  |  |  |  |  |  |  |  |  |  |  |  |  |  |  |  |  |
| AP8 | 0.53 | 0.59 | 0.68 | 0.86 | 0.96 | 0.93 | 0.97 | 1.00 |  |  |  |  |  |  |  |  |  |  |  |  |  |  |  |  |
| AP9 | 0.54 | 0.61 | 0.69 | 0.87 | 0.96 | 0.93 | 0.97 | 1.00 | 1.00 |  |  |  |  |  |  |  |  |  |  |  |  |  |  |  |
| AP10 | 0.40 | 0.48 | 0.61 | 0.83 | 0.96 | 0.91 | 0.96 | 1.00 | 1.00 | 1.00 |  |  |  |  |  |  |  |  |  |  |  |  |  |  |
| AP11 | 0.48 | 0.56 | 0.67 | 0.82 | 0.95 | 1.00 | 0.96 | 1.00 | 1.00 | 1.00 | 1.00 |  |  |  |  |  |  |  |  |  |  |  |  |  |
| AP12 | 0.50 | 0.57 | 0.65 | 0.85 | 0.96 | 0.93 | 0.97 | 1.00 | 1.00 | 1.00 | 1.00 | 1.00 |  |  |  |  |  |  |  |  |  |  |  |  |
| AP13 | 0.69 | 0.77 | 0.92 | 0.76 | 0.81 | 0.86 | 0.84 | 0.78 | 0.80 | 0.75 | 0.83 | 0.79 | 1.00 |  |  |  |  |  |  |  |  |  |  |  |
| AP14 | 0.55 | 0.61 | 0.78 | 0.71 | 0.83 | 0.81 | 0.80 | 0.75 | 0.77 | 0.77 | 0.79 | 0.81 | 1.00 | 1.00 |  |  |  |  |  |  |  |  |  |  |
| AP15 | 0.60 | 0.60 | 0.73 | 0.90 | 1.00 | 0.78 | 0.92 | 0.87 | 0.88 | 0.95 | 0.84 | 0.87 | 0.88 | 0.83 | 1.00 |  |  |  |  |  |  |  |  |  |
| AP16 | 0.46 | 0.46 | 0.58 | 0.83 | 0.91 | 0.71 | 0.85 | 0.80 | 0.81 | 0.86 | 0.75 | 0.87 | 0.77 | 0.91 | 0.92 | 1.00 |  |  |  |  |  |  |  |  |
| AP17 | 0.42 | 0.42 | 0.59 | 1.00 | 0.94 | 0.76 | 0.84 | 0.89 | 0.89 | 1.00 | 0.87 | 0.88 | 0.61 | 0.67 | 0.94 | 0.83 | 1.00 |  |  |  |  |  |  |  |
| AP18 | 0.80 | 0.80 | 0.80 | 1.00 | 0.80 | 1.00 | 0.80 | 1.00 | 1.00 | 1.00 | 1.00 | 1.00 | 0.67 | 0.40 | 0.80 | 0.25 | 1.00 | 1.00 |  |  |  |  |  |  |
| AP19 | 0.60 | 0.68 | 0.71 | 0.90 | 0.89 | 0.78 | 0.76 | 0.79 | 0.80 | 0.82 | 0.74 | 0.79 | 0.67 | 0.81 | 0.00 | 0.94 | 0.91 | 0.67 | 1.00 |  |  |  |  |  |
| AP20 | 0.58 | 0.58 | 0.54 | 0.83 | 0.87 | 0.75 | 0.81 | 0.84 | 0.85 | 0.90 | 0.79 | 0.85 | 0.65 | 0.78 | 0.87 | 0.87 | 0.88 | 1.00 | 0.95 | 1.00 |  |  |  |  |
| AP21 | 0.54 | 0.48 | 0.48 | 0.67 | 0.82 | 0.68 | 0.73 | 0.75 | 0.76 | 0.85 | 0.77 | 0.74 | 0.56 | 0.63 | 0.81 | 0.74 | 0.89 | 1.00 | 0.72 | 0.92 | 1.00 |  |  |  |
| AP22 | 0.54 | 0.61 | 0.76 | 0.80 | 0.88 | 0.93 | 0.84 | 0.87 | 0.87 | 0.92 | 0.92 | 0.87 | 0.89 | 0.97 | 0.87 | 0.88 | 0.88 | 1.00 | 0.88 | 0.85 | 0.75 | 1.00 |  |  |
| AP23 | 0.44 | 0.44 | 0.61 | 0.83 | 0.86 | 0.84 | 0.82 | 0.85 | 0.85 | 0.90 | 0.80 | 0.84 | 0.67 | 0.71 | 0.87 | 0.79 | 0.89 | 1.00 | 0.74 | 0.83 | 0.78 | 0.85 | 1.00 |  |
| AP24 | 0.41 | 0.47 | 0.61 | 0.78 | 0.96 | 0.86 | 0.90 | 0.93 | 0.93 | 1.00 | 0.92 | 0.92 | 0.69 | 0.75 | 0.95 | 0.87 | 1.00 | 1.00 | 0.86 | 0.91 | 0.87 | 0.87 | 0.92 | 1.00 |

**Supplementary Table S6:** Jaccard’s similarity coefficient measured among twenty-four accessions of Kalmegh (*Andrographis paniculata*)
