# Supplementary material for: Maximizing genetic gain through unlocking genetic variation in different ecotypes of kalmegh (Andrographis paniculata (Burm. f.) Nee)
Source: Front Plant Sci. 2022 Nov 7;13:1042222. doi: 10.3389/fpls.2022.1042222 (PMC9677111; doi:10.3389/fpls.2022.1042222)
Supplement: Supplementary file 10 [file Table_7.docx]

| **Source** | **degree of freedom** | **Sum of squares** | **Mean sum of squares** | **Estimated variance** | **Total variation in percentage** | ***P* value** |
| --- | --- | --- | --- | --- | --- | --- |
| **Among Pops** | 5 | 73.977 | 14.795 | 0.877 | 7% | 0.001 |
| **Within Pops** | 20 | 224.369 | 11.218 | 11.218 | 93% |  |
| **Total** | 25 | 298.346 |  | 12.096 | 100% |  |

**Supplementary Table S7**

**(A)**Summary statistics of AMOVA

**(B)** Percentage of variation explained by first three axes in PCoA using EST-SSR markers

| **Axis** | **1** | **2** | **3** |
| --- | --- | --- | --- |
| **%variation** | 28.66 | 16.18 | 11.21 |
| **Cum %variation** | 28.66 | 44.84 | 56.05 |
